# Two Novel Plant-Growth-Promoting *Lelliottia amnigena* Isolates from *Euphorbia prostrata* Aiton Enhance the Overall Productivity of Wheat and Tomato

**DOI:** 10.3390/plants12173081

**Published:** 2023-08-28

**Authors:** Manisha Parashar, Sanjoy Kumar Dhar, Jaspreet Kaur, Arjun Chauhan, Jeewan Tamang, Gajendra Bahadur Singh, Asyakina Lyudmila, Kahkashan Perveen, Faheema Khan, Najat A. Bukhari, Gaurav Mudgal, Mayank Anand Gururani

**Affiliations:** 1 University Institute of Biotechnology, Chandigarh University, Mohali 140413, Punjab, Indiasanjoy.r21@cumail.in (S.K.D.); jaspreet.r47@cumail.in (J.K.); gajendra.singh@cumail.in (G.B.S.); 2 Department of Biotechnology, Institute of Applied Sciences & Humanities, GLA University, Mathura 281406, Uttar Pradesh, India; 3 Laboratory for Phytoremediation of Technogenically Disturbed Ecosystems, Kemerovo State University, Krasnaya Street, 6, 65000 Kemerovo, Russia; 4 Department of Botany & Microbiology, College of Science, King Saud University, Riyadh 11495, Saudi Arabianajatab@ksu.edu.sa (N.A.B.); 5 Department of Biology, College of Science, United Arab Emirates University, Al Ain 15551, United Arab Emirates

**Keywords:** plant growth promotion, endophyte, biofertilizer, spurge, Euphorbiaceae, succulent, latex, tomato, wheat, productivity

## Abstract

Euphorbiaceae is a highly diverse family of plants ranging from trees to ground-dwelling minute plants. Many of these have multi-faceted attributes like ornamental, medicinal, industrial, and food-relevant values. In addition, they have been regarded as keystone resources for investigating plant-specific resilience mechanisms that grant them the dexterity to withstand harsh climates. In the present study, we isolated two co-culturable bacterial endophytes, EP1-AS and EP1-BM, from the stem internodal segments of the prostate spurge, *Euphorbia prostrata*, a plant member of the succulent family Euphorbiaceae. We characterized them using morphological, biochemical, and molecular techniques which revealed them as novel strains of Enterobacteriaceae, *Lelliotia amnigena*. Both the isolates significantly were qualified during the assaying of their plant growth promotion potentials. BM formed fast-growing swarms while AS showed growth as rounded colonies over nutrient agar. We validated the PGP effects of AS and BM isolates through in vitro and ex vitro seed-priming treatments with wheat and tomato, both of which resulted in significantly enhanced seed germination and morphometric and physiological plant growth profiles. In extended field trials, both AS and BM could remarkably also exhibit productive yields in wheat grain and tomato fruit harvests. This is probably the first-ever study in the context of PGPB endophytes in Euphorbia prostrata. We discuss our results in the context of promising agribiotechnology translations of the endophyte community associated with the otherwise neglected ground-dwelling spurges of Euphorbiaceae.

## 1. Introduction

Crop productivity, in terms of quality and quantity attributes, is globally challenged by multiple factors classified into abiotic and biotic stressors. Abiotic factors like drought, salinity, freezing, and unfavorable soil profiles affect crop physiology and reduce optimum growth. The majority of these factors in conjunction with the globally occurring climate change scenarios corroborate commercial harvest losses [1,2]. Mitigation of these challenges has involved conventional breeding approaches, newer transgenic crops, silviculture, crop rotation, and mixed farming approaches; intensive research is also ongoing into high-precision farming interventions like robo-farming, satellite farming, IOTs, and automated farming with unmanned vehicles, as well as developments of artificial intelligence platforms incorporated into agricultural practices [3,4,5,6]. These aim to maximize both the quality and quantity of crop harvest, yet also with the added focus on maintaining low carbon footprints and sustainable use of water, land, and energy resources [7,8,9,10,11].

Attempting to fulfill most of these aims, basic and applied biotechnology research communities have variously focused their attention on resilient underutilized crop models, weeds, and other wild plants as well as their associations with microbionts [12,13,14,15,16]. Many of these microbes offer applications as biofertilizers, bioinoculants, biocontrol, and bioremediation agents and as a source of a plethora of value-added bioactives for use in agriculture, pharmaceutics, palliative care, and probiotics [17,18,19,20,21,22,23]. Roadside weeds, including those from the Euphorbiaceae family, are often overlooked in agricultural research due to their perception as undesirable and invasive plants. These plants are commonly found growing in disturbed areas like roadsides, abandoned lots, and other neglected spaces. As a result, they are typically considered nuisances and not given much attention in terms of agronomic value. Many spurges in Euphorbiaceae are succulents, xerophytic, which easily grow on gravel pediments and rocks, and remarkably are unperturbed from mild to heavy public encroachments and walkover-bruises. Yet many possess the ability to grow on soils with limited water and low or no fertility, especially those in many technogenic locations burdened with disproportionate loads of heavy metals and other generally plant-unfriendly constituents. These wonderful resilience assets are worth noting. Scientific investigations are fairly negligible on how these spurges resist extreme edaphic conditions combined with extreme weather (with intense heat, freezing), grazing, insect herbivory, etc. However, it is not surprising that a few endemic species like *Euphorbia jodhpurensis* are considered to be threatened [24,25]. Amongst the predominant and ubiquitous spurges [26] is the sandmat spurge, *Euphorbia prostrata* (EP). This species comprises slender branches forming a peculiar rounded mat-like soil overlay. They can be seen easily growing wildly over cemented pavements, desert hot sands, and remarkably, unfavorable industry-intense sites. EP is also known to easily thrive with high soil salinity [27] and with hyperaccumulation of heavy metals, probably due to relatively higher biosynthesis of long-chain phytochelatins [28]. A recent study on its extracts proclaims it to be an eco-friendly corrosion inhibitor for steel [29].

The literature documents only these few investigative leads to date into EP. Other than this, leads into microbial associates specifically of EP have largely been ignored compared to reports on other Euphorbia spurges. Of the few examples, *Byssochlamys spectabilis* reported from EP from Sudan confers cytotoxicity toward the MCF7 breast cancer cell line [30]. Reports on microbial endophytes in other Euphorbia spurges include, for example, some fungal endophytes of pharmacological values [31,32] and biocontrol potentials against crop diseases [33,34]. *E. milli* are reported to variously influence hormone balance and pollution-absorptive features of the host [35,36], and another PGP endophyte from *E. milli* improves salinity tolerance in maize [37]. Another *E. milli* fungal endophyte, *Chaetomium ovatoascomatis*, shows antimicrobial efficacy [38] and a few laccase-producing fungal endophytes are also known [39]. Other than this, from *E. hirta*, few antimicrobial endophytes [40,41], an antioxidant-producing fungus *Nigrospora sphaerica* (EHL2) [42,43], and a hepatoprotective *Archaetomium* sp. are reported [44]. An *Aspergillus japonicus* EuR-26 strain of fungal PGP endophyte from *E. indica* was shown to improve the growth of soybean and sunflower under heat stress [45]. A fungal endophyte, *Chaetomium globosporum*, of *E. humifusa* has been shown to synthesize anti-phytopathogenic compounds [46]. Similarly, *Cladosporium oxysporum*, an endophytic fungus isolated from *E. bupleuroides*, has been shown to carry potential as a biocontrol agent against black bean aphids [47]. Still, very few groups discuss the abiotic stress tolerance attributes of Euphorbia spurge-associated microbionts and/or their translational interventions in crops [48].

At the outset, our group specializes in investigating resilient plants and/or their inherent microbial associations, aiming to translate and interpret their bioprospects into sustainable, eco-friendly, and cost-effective agricultural crop productivity [49,50,51,52,53,54,55]. In this study, we characterized two novel PGP bacterial endophytes from in vitro tissue culture attempts using *Euphorbia prostrata* (EP) and extensively investigated their ability to promote plant growth and productivity in agricultural crop models.

## 2. Results

### 2.1. Isolation of AS and BM from Tissue Culture Trials of E. prostrata (EP)

**Figure 1:**
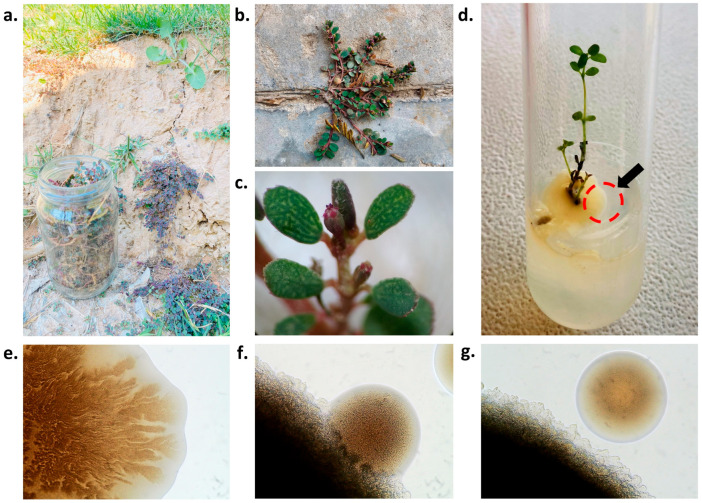
**The sandmat spurge, *E. prostrata* (EP), and its bacterial isolates.** Wildly growing EP, in (**a**), on garden soil and, in (**b**), over a cemented pavement; in (**c**), floral characteristics identifying EP (see text); in (**d**), a peculiar bacterial growth forming media overlay surrounding the stem-internodal explant, the latter responding in shoot growth; in (**e**–**g**), light microscopy observations (at 100× magnification) of the various sites over an NA plate streaked with a loopful of the mixed culture (as shown in (**d**)), where (**e**) shows a mixed culture (at mother streak), (**f**) with two distinctly morphed bacterial growths co-cultivated at a site on NA; (**g**) shows a site on NA with separately growing morphotypes. Note in (**e**–**g**)**,** BM shows swarming growth while AS shows smooth round colonies.

To explore the in vitro cultivability of EP for many bioprospecting studies, we collected plant samples from within Chandigarh University, at nearby construction sites, and/or from cemented pavements (Figure 1a,b). EP could be well differentiated from other spurges in its vicinity based on its floral characteristics (Figure 1c) [56,57,58]. In the various ongoing attempts with in vitro tissue culture of EP in our laboratory, we frequently observed contamination of in vitro established stem-internodal explants with a peculiar off-white bacterial growth, which with time developed a media-overlay surrounding the base of the growth-responsive EP explants (Figure 1d). This bacterial growth however did not affect the EP explant regeneration, although it conferred a week-early shoot emergence compared to those from the other aseptic EP tissue culture replicates. Curiosity-driven, we inoculated a loopful culture from this media-overlay onto nutrient agar (NA) plates and observed the colony characteristics. A close light-microscopy-assisted evaluation revealed this as a mixed culture of two putative morphologically distinct bacterial colonizers (Figure 1e–g): one had a smooth texture and rounded colonies (hereafter isolate AS), while the other exhibited rapidly expanding swarms with irregularly shaped colonies (hereafter isolate BM). Both AS and BM isolates could be seen as compatible, growing together without any zone of antagonism/inhibition (Figure 1e–g).

### 2.2. Morphological and Biochemical Characterization of AS and BM Isolates

Various morphological and biochemical characterizations (Table 1) defined the isolates AS and BM as Gram-negative, rod-shaped bacteria. Both isolates tested positive for catalase, methyl red, indole, citrate utilization, and motility assays and ferment sucrose, lactose, and dextrose. BM could distinctly also ferment glucose. Both isolates, however, remained negative in activities for pectinase, lipase, cellulase, and amylase.

### 2.3. Morphology of AS and BM under Scanning Electron Microscopy

**Figure 2:**
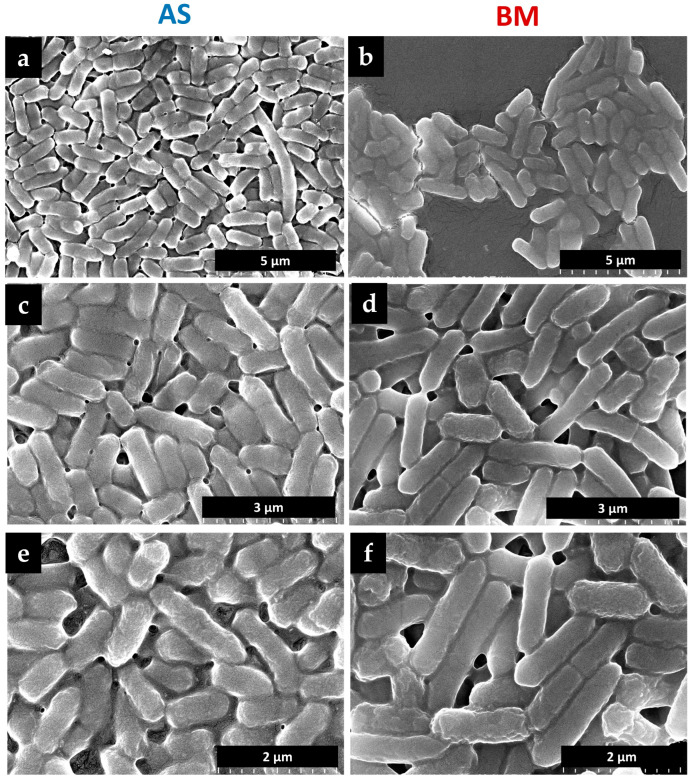
**SEM micrographs of the AS and BM isolates from the EP plant.** In the figure, panels form (**a**–**f**): panels (**a**,**c**,**e**) show morphological details of AS while panels (**b**,**d**,**f**) show details of BM (at various scales as shown in panels). Note BM with slightly bigger-sized rods than AS at a particular magnification.

Fe-SEM uncovered structural details of the two EP-isolated putative bacterial cultures. Both AS and BM appear as rod-shaped bacteria (Figure 2), with BM exhibiting a relatively but only slightly bigger size than the isolate AS. There were no other peculiar distinguishable characteristics under SEM analyses for these two isolates.

### 2.4. Antibiotic Sensitivity of AS and BM Isolates

Both AS and BM shared similar antibiotic sensitivity profiles with susceptibility to trimethoprim, spectinomycin, tetracycline, and gentamicin and resistance to streptomycin, penicillin, chloramphenicol, and ampicillin (Table 2). With these results, bacterial cultures were further stocked under selection pressure with the appropriate antibiotic (ampicillin).

### 2.5. Molecular Characterization of AS and BM Isolates

**Figure 3:**
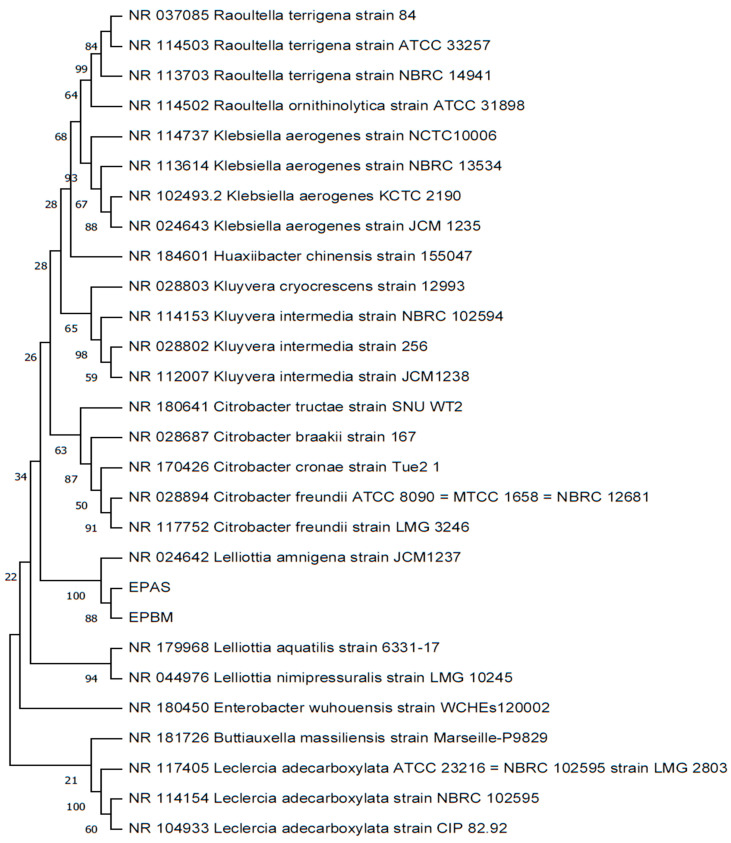
**Phylogenetic tree of AS (EPAS) and BM (EPBM).** Neighbor-joining phylogenetic tree constructed using partial 16S rRNA gene sequences of strain AS (1383 bases; GenBank accession OR342320) and strain BM (1484 bases; GenBank accession OR342321) illustrated the evolutionary relationship with their closely related bacterial strains. The numbers on the branches indicate bootstrap values derived from 1000 resamplings.

After phylogenetic analysis was conducted using the 16S rRNA gene sequences of isolates AS (1383 bases; GenBank accession number OR342320) and isolate BM (1484 bases; GenBank accession number OR342321), the neighbor-joining tree revealed a close relationship between these two strains and *Lelliottia amnigena* strain JCM1237 (GenBank accession number NR_024642), indicating a shared common ancestor (Figure 3). The results from the sequence similarity search further corroborated these findings, with strains AS and BM showing the highest levels of 16S rRNA gene sequence similarity with *Lelliottia amnigena* strain JCM1237, at 99.86% and 99.31%, respectively. Remarkably, partial sequences of both strains AS and BM were found to be 100% identical to each other. Despite their genetic relatedness, strains AS and BM exhibited differences in morphology and biochemical characteristics. Apart from this, other phylogenetic tree construction algorithms, such as maximum parsimony, maximum likelihood, and UPGMA, yielded similar phylogenetic tree patterns, supporting the conclusion that both strains belong to the genus Lelliottia. Currently, the genus comprises species such as *Lelliottia amnigena*, *Lelliottia nimipressuralis*, *Lelliottia aquatilis*, and *Lelliottia jeotgali*, primarily sourced from aquatic environments and plants [59].

### 2.6. PGP Attributes with AS and BM Isolates

As shown before, while AS and BM appeared as a mixed consortium initially emerging from the EP explants, they did not affect the growth propensity of EP explants in culture but eased in vitro culturing regimes with EP PTC (Figure 1). It was intriguing to test the plant-growth-promoting (PGP) characteristics possessed by each of the isolates, AS and BM. Table 3 highlights these properties in both isolates. Both the isolates were able to produce ammonia, fix N_2_, and biosynthesize the phytohormones IAA and GA. The highest activities in these parameters were detected only after 4 days of assay incubations. Zinc solubilization of a relatively high extent was only shown by the isolate BM. Both isolates, however, exhibited negative activity for potassium solubilization and the production of HCN and biofilm.

### 2.7. AS and BM Enhance Wheat and Tomato Crop Productivity

Although the outcomes of the above PGP assays vouch for the prospective potentials of *Lelliottia amnigena* isolates, for further translational merits, a sound validation of these potentials on model crop species is mandatory. Therefore, we variously tested the effects of AS and/or BM on commercially available wheat (PBW343) and tomato (Sakura F1 super Arjun) varieties under in vitro, ex vitro, and controlled field trial settings.

#### 2.7.1. Enhanced Growth of Wheat and Tomato Seeds under In Vitro Seed Priming with AS and BM

**Figure 4:**
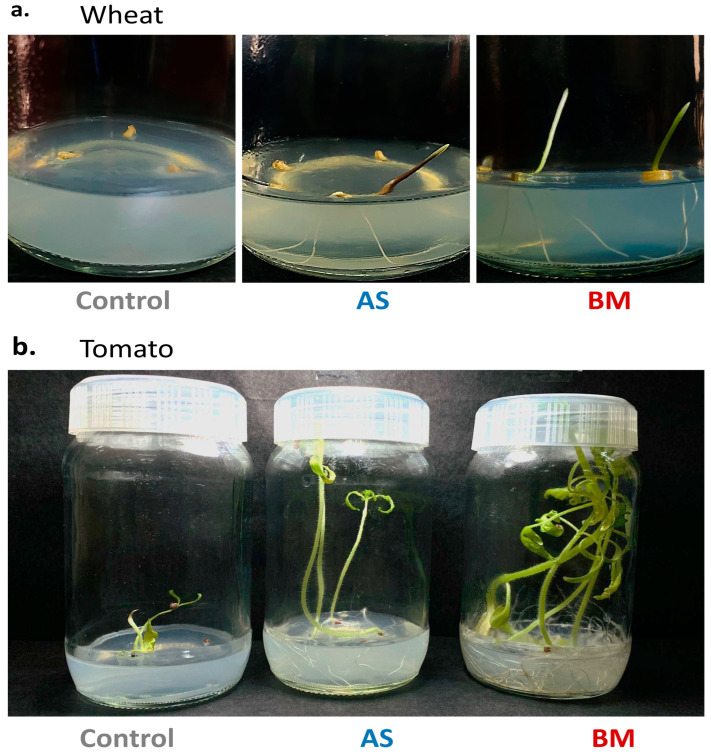
**In vitro wheat and tomato seed priming with AS/BM.** In (**a**), wheat seedlings; in (**b**), those of tomato on the 3rd day after in vitro establishment. Note that most of the primed wheat seeds germinated well while none in the control treatment could (shown in (**a**)). Similarly, priming effects were more pronounced with enhanced seedling stature than those in control sets (shown in (**b**)).

Surface-sterile wheat and tomato seeds (in separate trials) were primed overnight with clarified and filter-sterile spent supernatants from NB-raised inocula of AS and/or BM. Seeds were established over MS media in sealed PTC jam bottles and were allowed to grow under PTC room conditioning (see materials and methods). These AS/BM priming treatments (with clarified supernatants) favored seed germination response in both wheat and tomato trials (Figure 4a,b). Specifically, in trials with wheat seeds, AS/BM priming sets exhibited overall enhanced seed germination with plumule emergence within 3–3.5 days and with an overall germination response of ca. 95%. This, in control sets, however, could be observed only at 4–4.5 days and with a drastically lower extent (of about 75–80% germination). Similarly, in the case of tomato, priming significantly enhanced seed germination (with overall germination of 100% at about 6–7 days) as compared to the control treatments (with 70% evident by 8–8.5 days). Later effects in both tomato and wheat seedlings evidenced enhanced plant stature with well-formed foliar appendages which were easily distinguishable from plants under control treatments. Our results reflect that the PGP attributes in effect from the above seed-priming treatments (with either AS or BM) could be transferred to the crop plants. Moreover, BM seems more PGP-effective for seedling growth in all trials (Figure 4), in line with the PGP attributes assayed before (Table 1).

#### 2.7.2. AS/BM-Assisted Seed Priming Enhances Wheat Ex Vitro Growth

**Figure 5:**
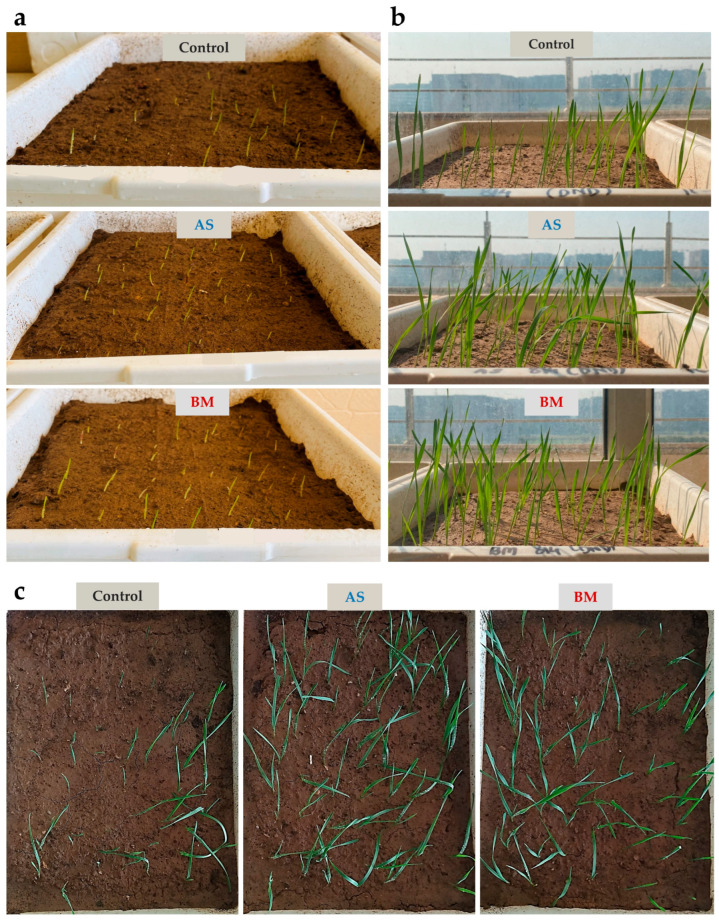
**Ex vitro trials with wheat seed pretreatments with AS/BM.** In (**a**), seed germination instances captured at 3.5 days post-sowing in all experimental sets (AS, BM, and control treatments), which in (**b**,**c**) are further captured to document seedling response in the representative trial(s).

**Figure 6:**
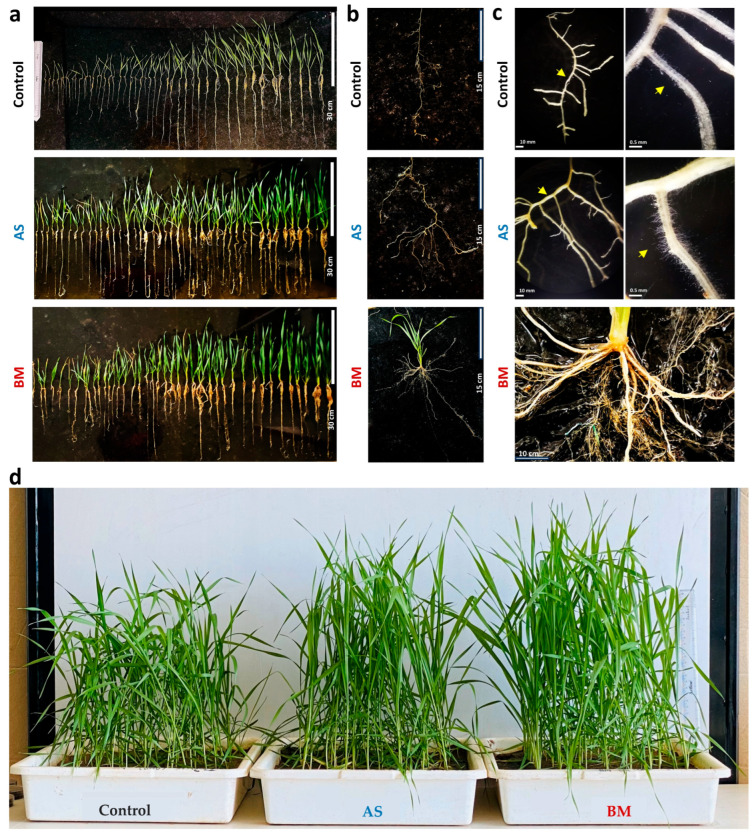
**Drain-out observations for ex vitro wheat pretreated with AS/BM.** In (**a**), lineups of all surviving plants from one of the representative tray trials with experimental sets of control and AS/BM treatments; in (**b**), representative root stature in the three experimental sets, note the drastically different root-branching patterns (control: slender; AS: tip tufts; BM: high adventitious root density at root–shoot interface); in (**c**), more peculiar close-up featuring putative PGP effects in AS/BM-assisted sets as compared to the control group; in (**d**), plants at height gradients probably documenting effects from PGP potentials of AS and BM, supporting the results in PGP assays (Table 3). Images scaled with bars as shown in panels where relevant.

**Figure 7:**
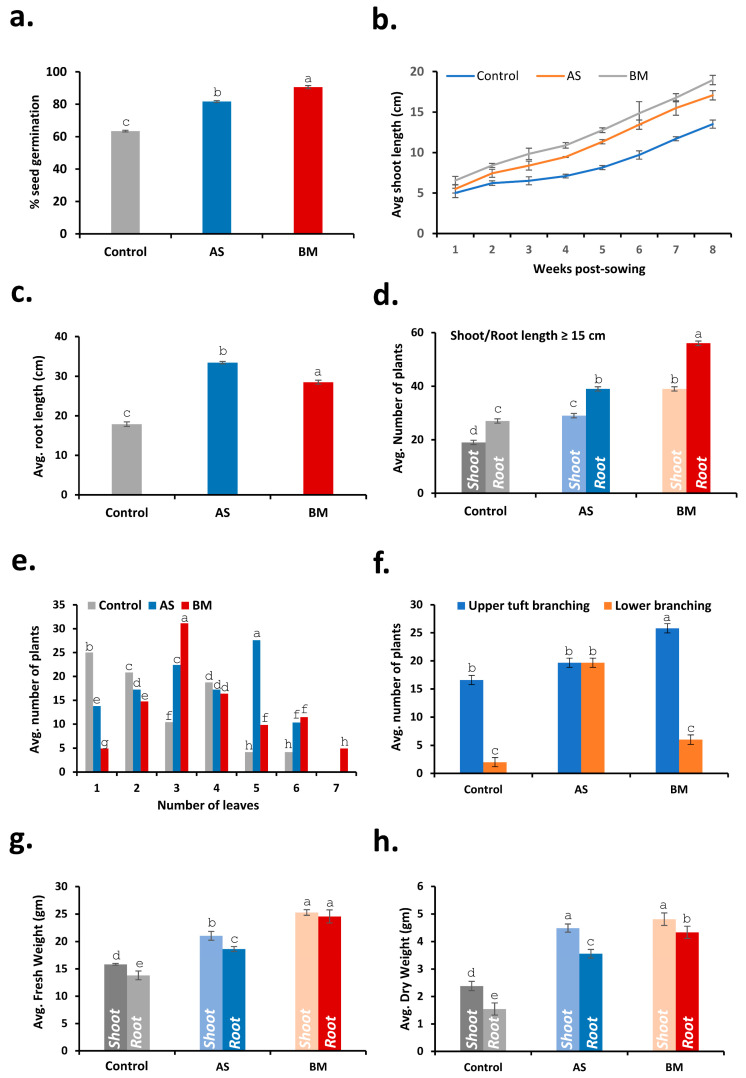
**Drain-out morphometrics and measured growth parameters for ex vitro wheat pretreated with AS/BM.** Measured effects compared amongst experimental treatments carried out in ex vitro settings (as also observed in Figure 5 and Figure 6); (**a**) depicts seed germination profiles; (**b**) depicts increments in shoot length with time. Post-drain-out measurements are shown in (**c**–**h**), where (**c**) shows average root length, (**d**) depicts variations along sets in the context of numbers of shoots and roots beyond a 15 cm margin, (**e**) shows another peculiar distinguishment based on multiples of plants with higher leaf counts, (**f**) shows number of plants with peculiar tip tuft (=predominant lower root branching, pronounced in AS) and adventitious roots (=predominant upper tuft branching, pronounced in BM), and (**g**,**h**) depict these shoot and root stature differences in terms of fresh and dry weights, respectively. If two variables have different letters above their bars, they are significantly different (*p* ≤ 0.05) as derived using the Tukey’s test.

Outcomes from in vitro testing of AS/BM-assisted seed priming of wheat seeds compelled us to investigate whether these PGP attributes in AS/BM would also benefit wheat growth under ex vitro settings. To do this, following from the above trials, ex vitro trials involved a CRD using a set of 60 healthy seeds of the same wheat variety which were primed with freshly grown overnight cultures of AS and/or BM (in NB as mentioned before) and sown (2 cm deep) in autoclaved garden-soil bedding established over plastic trays (see materials and methods for details). Every week after sowing, plants in the AS/BM-primed seed trays were booster-dosed with an overnight-grown fresh inoculum of AS and/or BM. Control trays (with wheat seeds devoid of seed-priming treatment) were similarly only wetted with an equivalent volume of NB. These ex vitro tray trials were run for 12 weeks to record seed germination profiles, seedling morphometrics, physiological growth, and antioxidant profiles, which are shown in Figure 5, Figure 6 and Figure 7. As seen with in vitro trials (Figure 4), here the overall seed germination rate was significantly enhanced in the case of AS/BM-primed seed trays (plumule emergence within 2–2.5 days after sowing), and both cases also exhibited an overall higher germination extent (AS, 80%; BM, 90%) compared to control treatments (with about 60–65% germination, Figure 5a) on the third day after sowing (Figure 7a). Early germination with seed-priming treatments indicates an overall enhanced plant growth rate which is also exemplified by increased shoot growth morphometrics (length of shoots) with time (Figure 5b,c and Figure 7b). Morphometric measurements recorded after soil drain-out from tray trials at the 12th week (3 months) post-sowing provided further strong indications of PGPB-assisted wheat growth (Figure 6a–d and Figure 7c–h). These drain-out data revealed significant increments in the overall length and numbers of shoots, roots, and root branches and the root girth in wheat seedlings raised from the AS/BM treatments compared to those raised in control treatments (Figure 6a–d and Figure 7c–h). Nonetheless, the overall count of shoots and roots above a set margin (≥15 cm) were significantly higher in seed-priming–booster-dosing-raised seedlings than in the case with control treatments (Figure 7d). Another peculiar observation from the drain-outs could be recorded specifically regarding root stature in all trials. Most roots in control treatments (above 70–75%) were slender, with one main root showing smaller branching along the length (Figure 6a–c). In the plantlets from AS-primed–booster-dosed treatments, most main roots (about 80–85%) showed overall higher branching extents along their length and were more pronounced at the tip, while those in BM treatments surprisingly showed adventitious rooting and root branching at the shoot–root interface (Figure 6b,c). These differences in wheat root stature amongst the experimental sets were also exemplified by their overall fresh/dry weight measurements throughout the various trials (Figure 7g,h).

#### 2.7.3. AS/BM-Assisted Seed Priming Also Enhances Ex Vitro Growth of Tomato

**Figure 8:**
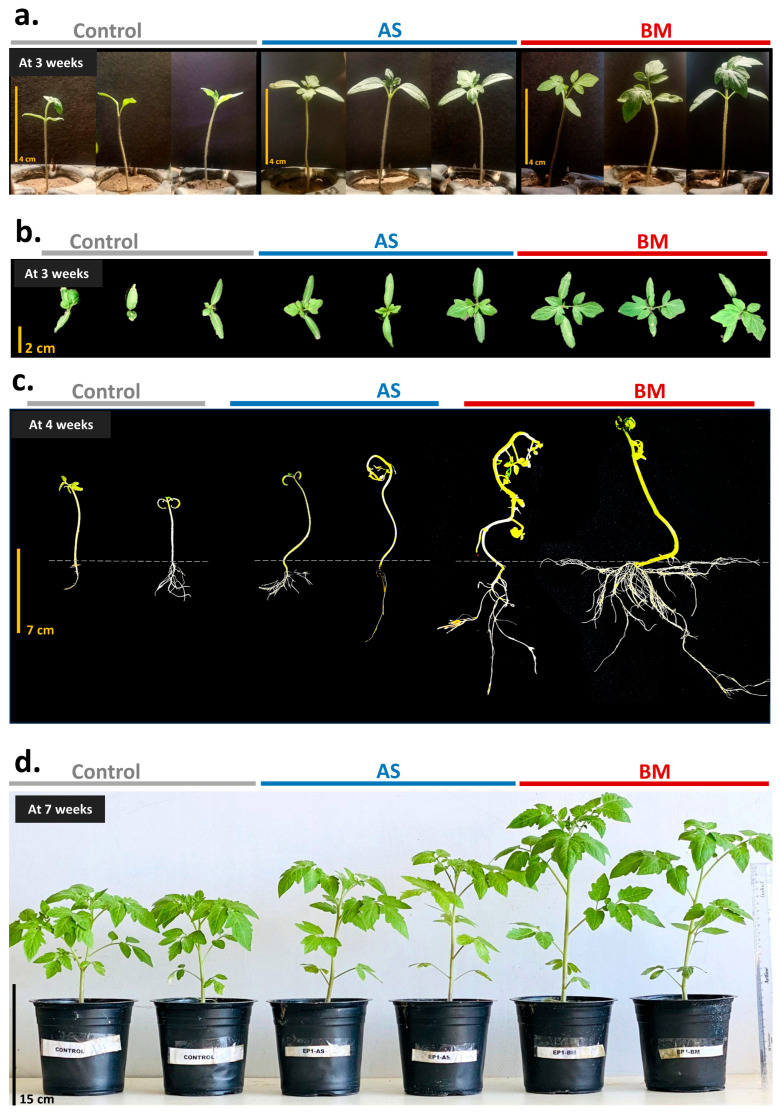
**Ex vitro trials with tomato seed pretreatments with AS/BM.** Images captured at various time intervals (as shown in the top left in each panel) after seed sowing in tray pots, where (**a**) shows representative effects in seedlings in various experimental sets distinguishable in height above the soil; (**b**), similarly, shows an image captured at the same frame to compare foliage spread (area) to compare the effect of AS/BM treatments; (**c**) shows drain-out representatives revealing PGP effects from AS/BM on the whole seedling stature. Note the drastic effects of root architecture in the case of BM sets; and (**d**) shows a representative image from alternate extended trials with seedlings transferred from tray pots to big-sized pots to reproducibly showcase plant height increments in the effect of AS/BM treatments. Images scaled to bars are shown in each panel.

**Figure 9:**
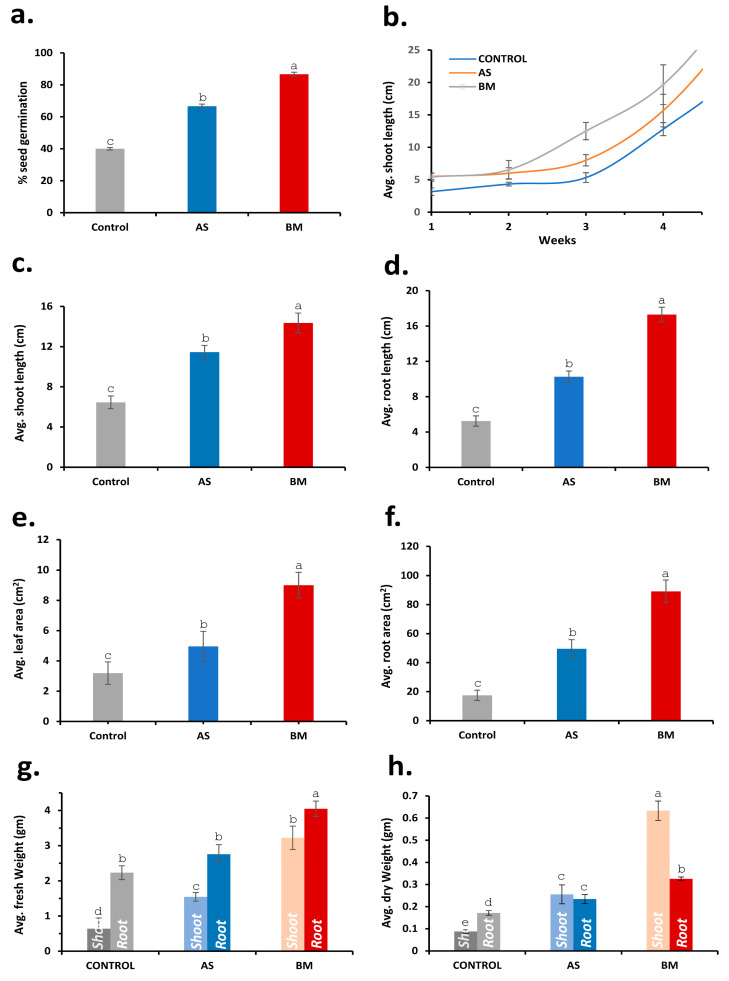
**Growth profiling and morphometrics followed in ex vitro trials with tomato**. In (**a**), seed germination; (**b**), shoot length increments with time; (**c**), shoot length at a month; (**d**), root length post-drain-out after a month; (**e**), leaf (=photoreceptive area) area (overall foliar spread per plant in a month); (**f**), root spread per plant; (**g**,**h**), fresh and dry weights of plant shoot and root sections on overall per-plant basis. For details, see text and materials and methods. If two variables have different letters above their bars, they are significantly different (*p* ≤ 0.05) as derived using the Tukey’s test.

As seen with wheat (Figure 5, Figure 6 and Figure 7), the effect of AS/BM-assisted seed priming in tomatoes was also verified in seed germination and growth under ex vitro conditions (Figure 8 and Figure 9). About 40 healthy seeds were followed with separate seed-priming treatments with either AS or BM (see materials and methods) and later sown respectively over autoclaved garden soil in nursery plastic tray pots (see materials and methods). As with wheat trials, dosing interventions in control tomato tray sets were replaced with an equivalent volume of NB. Alternatively, in the follow-up trials after these, all plants for each experimental set were also transferred to individual big pots when shoots in control sets reached a height of ≥12 cm. For these tray and follow-up pot trials conducted for up to 5 months under glass-house conditioning (see materials and methods), plant morphometrics and physiological growth profiles were analyzed. Like under in vitro tomato trials, AS/BM-seed-priming-assisted seed germination in tomatoes under an ex vitro regime (Figure 9a) was also found to be significantly quicker (plumule emergence within 4.5–5 days for both AS and BM) compared to seed germination in control trays (6.5–8 days for plumule emergence). Figure 8 depicts the plant growth promotion effects from AS/BM treatments with an apparent enhancement in the number of leaves, roots, branching, and other characteristics. At about 4 weeks post-sowing, seed-priming-raised plants showed significant increments in both height and foliage spread compared to control tray plants, supporting the previous results of in vitro trials. AS/BM-raised plants showed a distinct foliar appearance compared to controls, which was a more apparent and vivid distinguishment in BM tomato plants, seen with more leaflets (Figure 8b). These increments in foliar appendages (with seed-priming treatments) in trays (Figure 8a–c) were sustained further following pot-transfer regimes in separate trials (Figure 8d). Healthy foliage indicates better photoautotrophic growth following the fortification of plants either from soil or inherently from bacteria, improving physiological growth.

#### 2.7.4. Physiological Growth Profiling Supports AS/BM-Assisted Enhanced Ex Vitro Wheat and Tomato Growth

**Figure 10:**
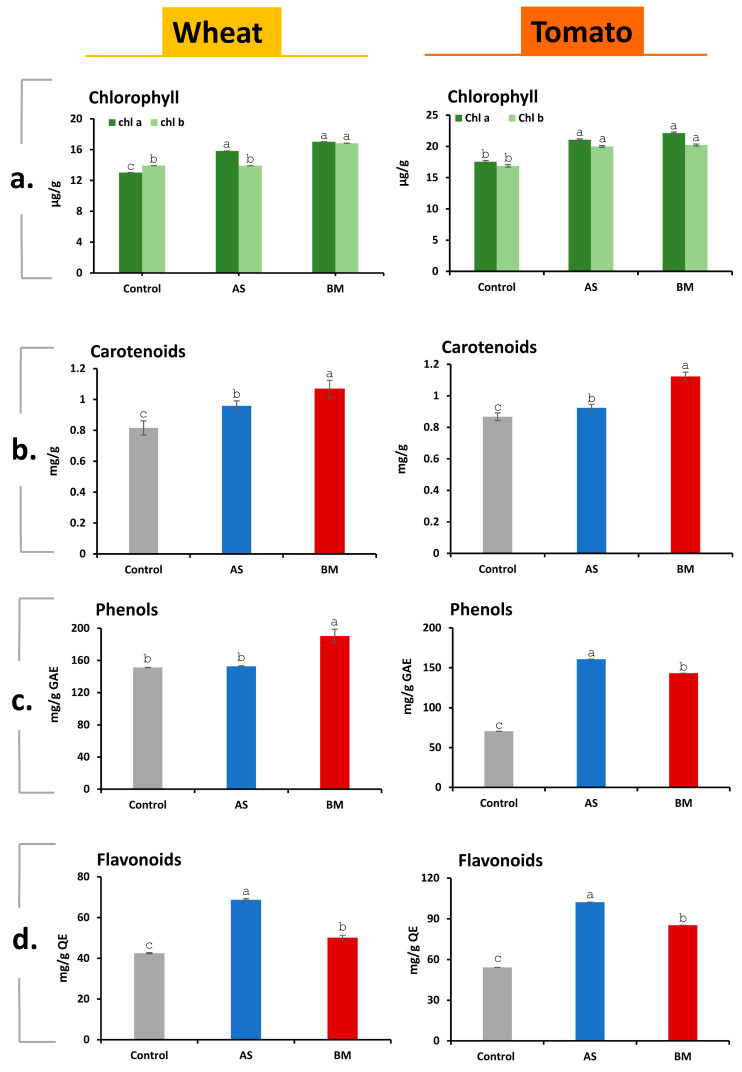
**Physiological growth profile of ex vitro trialed wheat and tomato crops under AS/BM treatments.** In (**a**), total chlorophyll; in (**b**), total carotenoids; in (**c**), total phenols; and in (**d**), total flavonoids estimated from leaf tissues of respective crops grown under seed priming and booster dosing with AS/BM isolates of EP. Each dataset presented here represents the means of three independent experiments. In each experiment, five replicates were randomly sampled for each of the independent control, AS, and BM treatments. The values obtained for phenols and flavonoids are expressed relative to their gallic acid (GAE) and quercetin (QE) equivalents, respectively. If two variables have different letters above their bars, they are significantly different (*p* ≤ 0.05) as derived using the Tukey’s test.

To verify the growth enhancement in ex vitro trialed wheat and tomato seedlings under separate treatments with either AS or BM (Figure 9), various standard physiological plant growth parameters were analyzed, including total chlorophylls, carotenoids, phenols, and flavonoids. The overall profiles of these parameters showed increased levels in both wheat and tomato seedlings (Figure 10). Improvements in the photosynthetic pigment profile would naturally contribute to the overall productive physiology of the plants [60]. Phenols play a crucial role in protecting plants from abiotic stressors by deactivating reactive oxygen species (ROS) and facilitating antioxidation [61]. As a result, the total phenolic content significantly increased in both wheat and tomato seedlings treated with either AS or BM compared to the control group. Moreover, total flavonoids may include specialized secondary metabolites that accumulate to counteract various plant stresses [62].

#### 2.7.5. Field Trials Validate PGP Prospects with AS and BM on Wheat and Tomato

**Figure 11:**
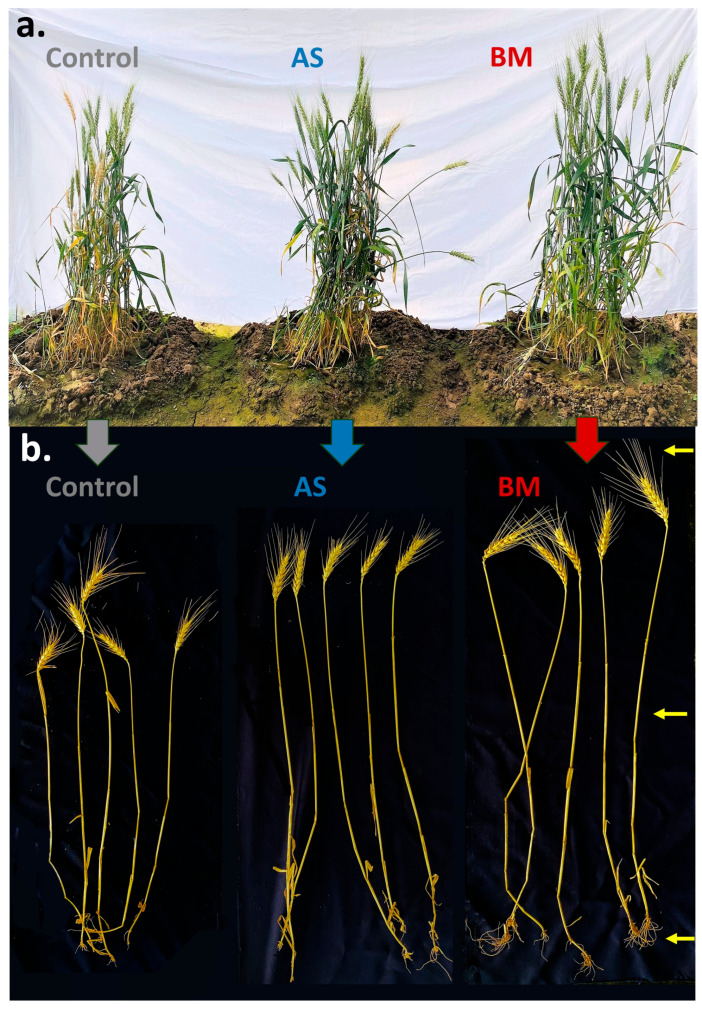
**Field-grown wheat under AS/BM treatments.** In (**a**), field growing wheat; in (**b**), pulled-out shoots for morphometrics and harvest yield profiling. Note the overall difference in shoot height in plant lots amongst various experimental treatments (control, AS, and BM).

**Figure 12:**
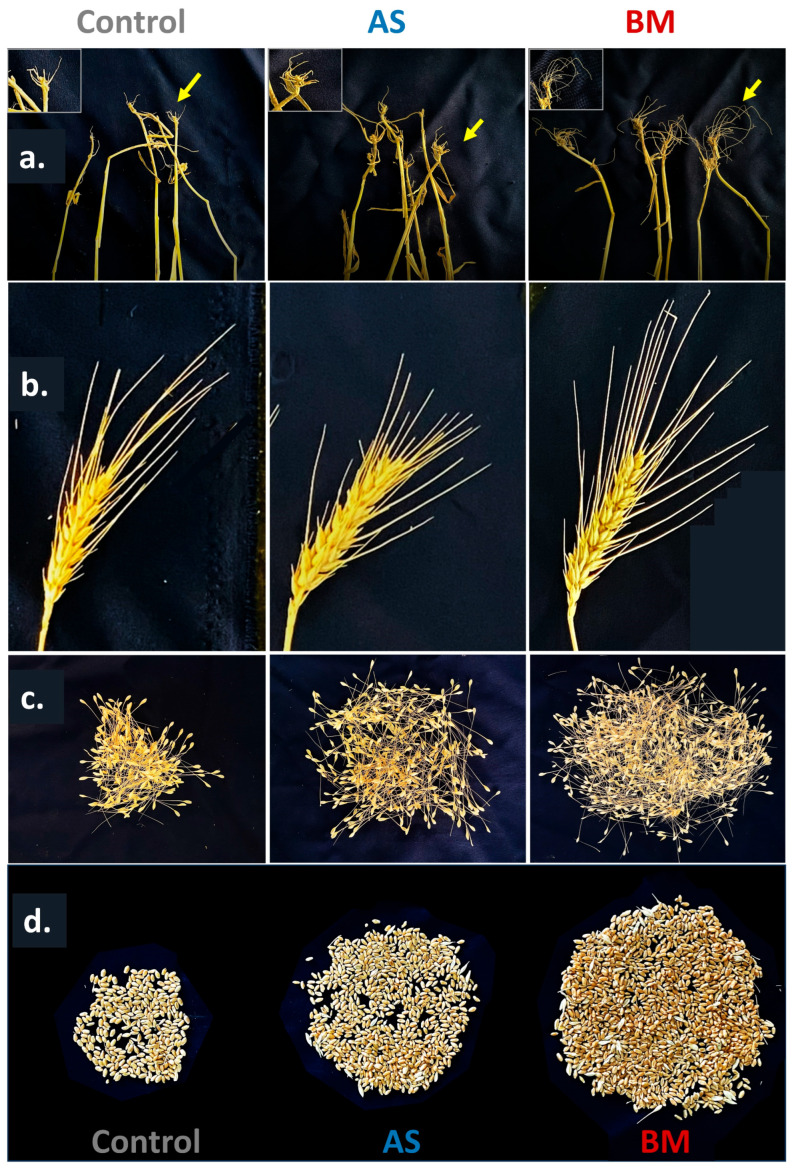
**Observations of post-field-trialed wheat pull-outs.** In (**a**), root stature (zoomed-in insets); in (**b**), grain heads/spikes; in (**c**), spikelets with grains sorted out; in (**d**), overall grain yield representatives for each experimental treatment. Note the root length and adventitious features in AS and BM are higher than those in control sets (in (**a**)). Also, note the grain heads are higher in length (in (**b**)).

**Figure 13:**
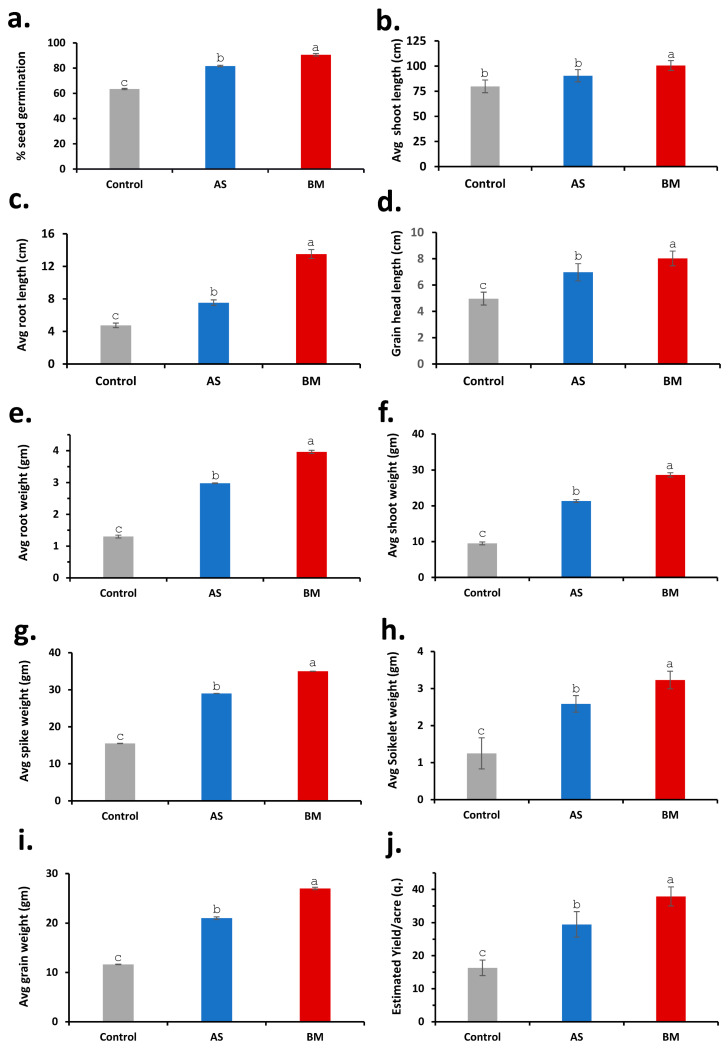
**Performance and yield at harvest stage of AS/BM-treated wheat under field trials.** In (**a**), seed germination profile; in (**b**), shoot lengths**;** in (**c**)**,** pulled-out root lengths; in (**d**), grain head (spike) length; in (**e**), root weight; in (**f**), shoot weight; in (**g**), spike weight; in (**h**), spikelet weight; in (**i**); grain weight. Note the increased values in both AS and BM sets relative to controls. In (**j**), estimated acre-scale yield. All trials were performed thrice with three replicate plots following a CRD. Values depict the overall means of the three independently carried out trials with error bars jotted from standard deviations. If two variables have different letters above their bars, they are significantly different (*p* ≤ 0.05) as derived using the Tukey’s test.

**Figure 14:**
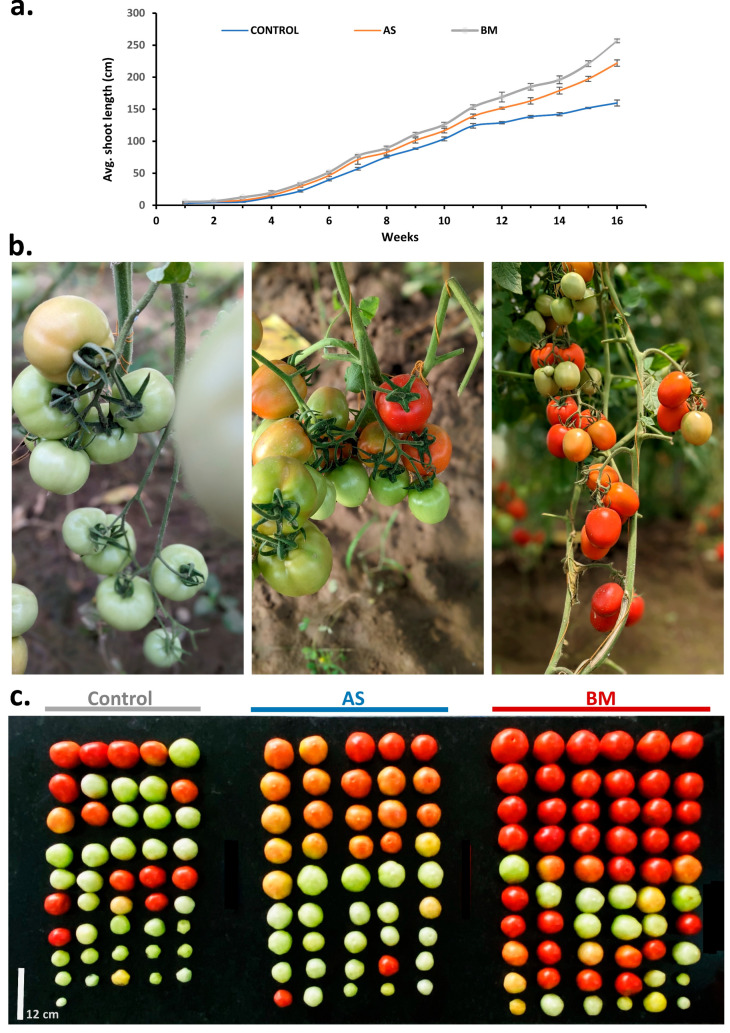
**Field trials with tomato under AS/BM treatments.** In (**a**), shoot length increments with time; in (**b**), fruiting in experimental sets at an instance; in (**c**), yield harvest per plant representative of the three independent trials. Note the overall number of fruits and their ripening proportion in each of the control, AS, and BM sets in (**c**).

**Figure 15:**
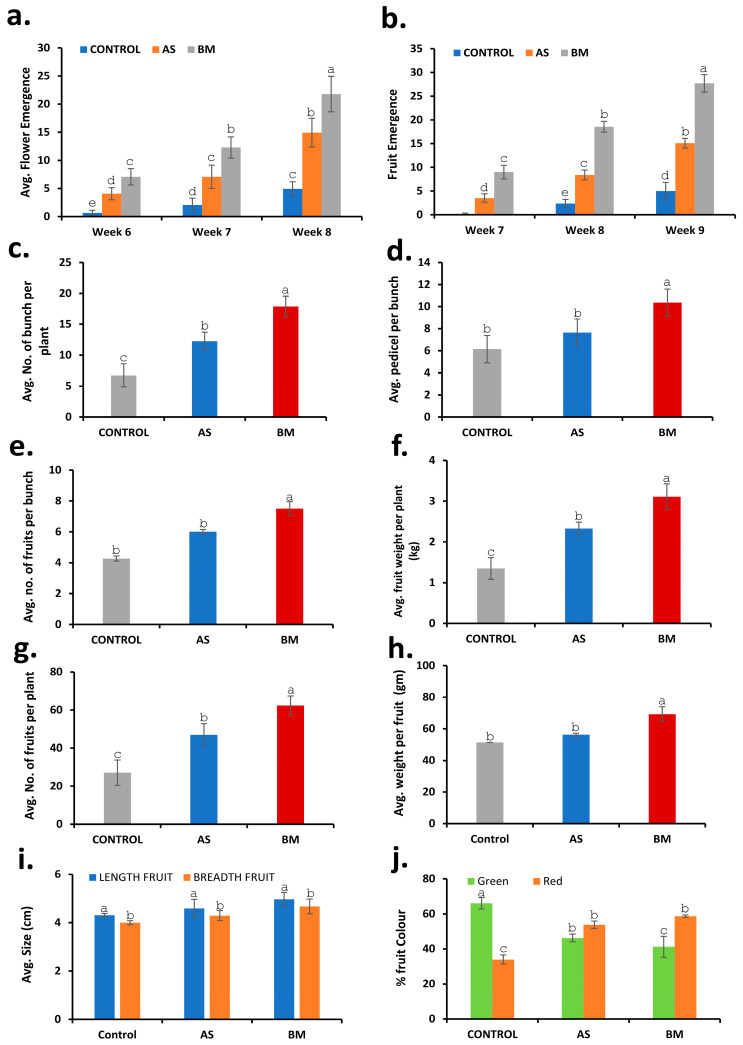
**Demographics and harvest data from field trials with tomato under AS/BM treatments.** In (**a**), flower (bud) emergence profile; in (**b**), fruit emergence; in (**c**), bunches per plant; in (**d**), pedicles per bunch; in (**e**), fruits per bunch; in (**f**), fruit weight per plant; in (**g**), number of fruits per plant; in (**h**), overall per-fruit weight; in (**i**); the average size of fruits; in (**j**)**,** the proportion of fruits either red or green (ripening parameter). Note the increased values in both AS and BM sets relative to controls. All trials were performed thrice with three replicate plots following a CRD. Values depict the overall means of the three independently carried out trials with error bars jotted from standard deviations. If two variables have different letters above their bars, they are significantly different (*p* ≤ 0.05) as derived using the Tukey’s test.

In vitro and ex vitro trials with seed-priming treatments on both wheat and tomato with either AS or BM indicated a significant enhancement of both morphometrics and physiological growth parameters (Figure 4, Figure 5, Figure 6, Figure 7, Figure 8, Figure 9 and Figure 10), which are well supported concerning the PGP potentials assayed before (Table 3). We wished to validate these potential effects in the field setting as well. Like before, AS- and BM-primed wheat and/or tomato seeds were sown over soil beds in the polyhouse; the latter consisted of farm soil without any treatment with fertilizers or any exogenous plant-growth-enhancing agents or any biocidal agents (see materials and methods). Booster dosing with AS/BM was accomplished using the soil drenching method for every seedling at weekly intervals post-sowing, and plants were irrigated with 5 L of tap water at an interval of every two days post-sowing. Results from the above field trials with both wheat and tomato cultivars have been presented here (Figure 11, Figure 12, Figure 13, Figure 14 and Figure 15).

Figure 11, Figure 12 and Figure 13 document various observations recorded for AS/BM-treated wheat grown under field trials. As seen with ex vitro wheat trials (Figure 5, Figure 6 and Figure 7), field-grown wheat crops followed with AS/BM seed priming also showcased a relatively enhanced overall shoot height above the ground (Figure 11a,b and Figure 13b). This could also be supported by an enhanced seed germination profile as also seen under in vitro and ex vitro trials (Figure 4a, Figure 5a, and Figure 7a). The observations in the field trials showed that the AS/BM-primed wheat seeds exhibited faster germination with an overall plumule emergence of about 80 and 90%, respectively, compared to only about 60% in control unprimed seeds (Figure 13a) within 3 days post-sowing. With time under field settings, shoot height increments followed similar trends as shown before with ex vitro trials (Figure 7b), such that significant differences in shoot height could be recorded in most plants in each experimental set at 13 weeks post-sowing (Figure 11a and Figure 13b). It was at this point that few plants in the control group indicated an onset of drying, indicating readiness for grain harvest (Figure 11a). However, the AS/BM-treated sets did not exhibit such maturity indications, which actually could only be seen a week after those in the case of controls. At the 16th week post-sowing, wheat crops in all the trials were pulled off to account for morphometrics and harvest yields. Furthermore, overall size increments under AS/BM priming treatments were more vividly documented with the spikes (head), stalk, and root region (Figure 12 and Figure 13) of the plants post-pull-out (Figure 11b). A close examination of root sections of pulled-out plants from each of the sets in control and AS/BM treatment groups (Figure 12a) revealed observations similar to those seen under ex vitro trials. Although secondary roots and branches thereof could have been damaged due to pulling out, the overall root architecture still could indicate a longer adventitious rooting feature in plants from the AS/BM treatment groups compared to only a few small stilt roots in the control-treated plants (Figure 11b, Figure 12a and Figure 13c). This result also indicated that AS/BM priming could have been attributed to better rooting with heightened strength keeping them largely less affected by pull-out maneuvers than the plants under the control treatment. This is also supported by differences in the root and shoot weight measurements amongst AS/BM priming and control treatments (Figure 13e,f). Furthermore, the spikes (grain heads) appeared slightly bigger (Figure 12b) with an overall higher length in plants under AS/BM treatments than those of the control group (Figure 13d). With a high germination rate in AS/BM-primed wheat, the percentage survival was also comparably improved all through the trial period of 4 months from sowing until crop harvest. Considerably, this, as well as the PGP effects, contributed to the overall plant productive growth including parameters related to spikes/spikelets and grain weight (Figure 12c,d and Figure 13g–i). Based on the area-wise productive grain yield in our trials, we estimated that in an acre-scale field sown with AS/BM priming and a booster-dosing regime, the commercial PBW343 variety may result in an average of about 30 and 40 quintals (for the AS and BM isolate treatments, respectively), compared to an estimated average yield of about 15 quintals without any exogenous treatment.

Similar to in vitro and ex vitro trials (Figure 8, Figure 9 and Figure 10), field-grown tomato plants also showcased enhanced plant growth characteristics and an overall productive fruit yield (Figure 14 and Figure 15). Precisely, as seen under in vitro conditions, seed germination showed a faster trend with enhanced shoot development all through the 16-week field trial with AS/BM-primed seeds compared to that in control groups (Figure 14a). Flower bud development could be seen by 5 weeks post-sowing in AS/BM-treated plants and was delayed by almost a week in the case of the control plants. Hence, under field trials with tomato plants, AS/BM treatments enhanced shoot development which was also followed by an overall enhanced flowering, which was estimated with their early emergence and greater multiples of flower buds per plant relative to the control treatments (Figure 15a). AS/BM treatments in turn also showcased a relatively early emergence of fruit appendages by the 6th week, and in the control group, the emergence of fruit appendages was sparingly witnessed only after a delay of 4–5 days (Figure 15b). Differences in experimental groups were also witnessed for fruit bunch multiples per plant and were more pronounced in the 9th week of the trial (Figure 15c). Pedicel formations per bunch also saw overall increments under AS/BM treatments (Figure 15d), which probably corroborated increments in emerging flower and fruit appendages (Figure 15a,b) on a per-plant basis as well as fruit numbers (Figure 15e) on a per-bunch basis. These latter results mark the productivity attributed to seed priming and booster dosing with AS/BM. In turn, in these cases, the overall yield by weight per plant (Figure 15f) as well as the number of fruits per plant (Figure 15g) both could be seen with drastic improvements. In line with these effects from AS/BM treatments, close examination revealed an overall increase in fruit biomass (Figure 15h), probably from a relatively upsized fruit body (Figure 14c and Figure 15i), as elucidated using fruit weight and morphometric profiling throughout the three experimental lots in each of the independent trials. Other than these, harvested fruits were analyzed visually for color profiles (Figure 14b,c). It is evident that AS/BM lots consisted of a bigger proportion of red tomatoes than those in the control group, where green-colored tomatoes were predominant (Figure 15j). No doubt, and as also seen in ex vitro trials, growth-promoting effects from BM treatments on tomato hold more promising potentials than those seen with AS treatments, while both are still more productive than effects seen in control groups without any pretreatments or booster dosing effects. These results are significantly remarkable in the context of greater harvest yields under tomato treatments with AS/BM priming and may offer upscaling benefits provided similar studies on scale-up cropping regimes.

## 3. Discussion

To tap bioprospects of resilient plants, our research theme is based on exploring resilient succulent plants and their connections with the endophytic microbial community [49,50,51]. Ground-dwelling spurges are a diverse group of plants belonging to the family Euphorbiaceae. Often ignored for their invasive occurrence as roadside weedy outgrowths, they have usually adapted to various ecological niches and are found in different habitats worldwide. While carrying out our tissue culture attempts with the sandmat spurge *Euphorbia prostrata* (EP), we isolated a mixed culture of two morphometrically, biochemically, and molecularly distinct endophytic *Lelliotia amnigena* isolates, AS and BM from the internodal EP explants. AS formed smooth colonies while BM showed swarming movements over nutrient agar.

The study resulted in PGP prospects that enhanced crop growth and productivity from lab- to field-tested regimes under seed-priming treatments with these EP microbial endophytes. These endophytes exhibited PGP effects and concomitant prospects in crops, which were more pronounced with the seed-priming-assisted treatments in cases with the BM isolate compared to the AS isolate and/or the control treatments.

Since its first discovery in the 1980s, *L. amnigena* has seen taxonomical rearrangements, moving from Enterobacter H3 into two subgroups of *Enterobacter amnigenus* and finally being introduced into a new genus *Lelliotia* within the family Enterobacteriaceae [63,64,65,66]. As per the literature and as we also document in this study, these *L. amnigena* isolates are motile, Gram-negative, rod-shaped bacteria [67]. Our two *L. amnigena* isolates, AS and BM, both show resistance to the broad-spectrum antibiotic chloramphenicol, the aminoglycoside-class antibiotic streptomycin, and two β-lactam antibiotics, penicillin and ampicillin (Table 2). There have been many anomalies related to antibiotic sensitivity in *L. amnigena.* It was generalized before that *L. amnigena* naturally resists second- and third-generation cephalosporins like cefoxitin, cefotaxime, and cefaclor [68]; later, certain strains demonstrated resistance to gentamicin, doxycycline, nitrofurantoin, and combinations of β-lactam/β-lactamase inhibitors such as amoxicillin/clavulanic [69]. However, these claims find a contradiction with a previous study [70] which suggested no natural resistance to cephalosporins including cefoxitin, but a reduced susceptibility to cefixime, cefpodoxime, and ceftibuten. Other than this, while several studies have indicated the presence of the *ampC* gene in *L. amnigena*, no detailed investigations have been conducted on the AmpC β-lactamase of *L. amnigena* [71].

As members of Enterobacteriaceae, *L. amnigena* strains have largely been considered as being of pathological concern; however, they have rarely emerged as a serious pathogen according to clinical and environmental records [59,65,72,73,74,75,76,77]**.** Amongst the *Lelliotia* species, *L. nimipressuralis* had been previously more reported in plants, while *L. amnigena* had been widely used as a marker for detecting food contamination [75]. It is only recently that many new *L. amnigena* isolates have been identified as causative of soft rot diseases in plants like potato [78,79,80] and onion [67]. Given their ability to thrive in the nutrient-limited conditions of drinking water reservoirs and lakes utilized for potable water supply, the hygienic significance of *Lelliotia* spp. becomes a matter of paramount concern for water regulatory bodies and providers [81].

In our study, the deemed-contaminating AS/BM isolates turned out to possess remarkable PGP properties which further were shown to potentially enhance growth and productivity parameters in wheat and tomato cultivars. In the context of the PGP effects of *L. amnigena*, only a few studies have reported on two wheat rhizosphere isolates, namely strain MSR-M49 and strain 15/1, both of which confer salinity tolerance attributes to wheat [82,83]. The MSR-M49 strain drastically differs from both our AS/BM isolates in producing an exopolysaccharide and is unable to fix N_2_. The strains AS and BM that we studied, however, were negative for any EPS-assisted biofilm activity (using the Congo red staining inference from EPS producers) and could positively fix N_2_ (Table 2). *Lelliotia* spp. are well adapted to survive in oligotrophic environments and have been isolated mostly from plants and water [59]. Our studies thus provide evidence on the plant-growth-promoting *L. amnigena* strains with their first reported occurrence as plant endophytes in *Euphorbia prostrata*, other than the previous knowledge of their reported presence only in the wheat rhizosphere [82,83]. It would be further intriguing to explore how some such *L. amnigena* strains associate and colonize in plants and their molecular biology of interactions that might variously offer biotechnological prospects. While both AS and BM isolates were found to be *L. amnigena* strains based on molecular characterization and were compatible for growth together, they still exhibited a few differences, such as those in their in vitro growth patterns, morphology under SEM, glucose utilization, and to some extent antibiotic sensitivity. They also differ in variously assayed PGP potencies, in imparting morphometric and architectural variabilities to roots and shoots in both wheat and tomato, as documented with various seed-priming treatments. These nuances offer a unique opportunity to uncover potential synergistic interactions between these strains. Understanding the mechanisms behind their coexistence could provide insights into microbial communication, cooperative behaviors, and the factors contributing to enhanced plant growth promotion. This could further lead to the development of more effective microbial consortia for improving crop productivity.

Although many *Lelliotia amnigena* isolates have been reported to cause soft rot in vegetable crops, the antagonistic potential of one variant against the fungal pathogen *Macrophomina phaseolina*, causing charcoal rot disease in chickpeas, is also reported [84]. Some reports have highlighted the capability of some *L. amnigena* strains to tolerate forms of pollution like high selenium in soil and high nitrates in water [72,85]. It is thus intriguing to further assess whether other PGP counterparts of *L. amingena*, including our EP isolates, AS and BM (individually and/or synergistically), would also possess these and other tolerance features and contribute to crop productivity under such environmental adversities.

Following our observations with EP in vitro PTC attempts, the two *L. amnigena* isolates, AS and BM, seemed to enhance the in vitro regeneration efficiency in EP. This productively circumvented the difficulty in establishing whole-plant regenerants from various aseptically maintained EP explants which otherwise appeared nonresponsive for any meristemoidal growth. The latter difficulty is otherwise commonly witnessed with many latex-bearing plants in Euphorbiaceae [86,87,88,89]. We wish to further ascertain the usability of these isolates (AS/BM) in achieving in vitro regeneration success in other difficult-to-propagate latex-bearing plants in Euphorbiaceae and other families.

We could variously document that seed-priming and booster-dosing treatment with either isolate (AS/BM) enhances overall morphometrics, physiological growth, and crop productivity profiles in commercially grown varieties of tomato and wheat (Figure 4, Figure 5, Figure 6, Figure 7, Figure 8, Figure 9, Figure 10, Figure 11, Figure 12, Figure 13, Figure 14 and Figure 15). Considering the results from our ex vitro trials with wheat and tomato seedlings, where individual treatments using AS or BM demonstrated growth enhancement (Figure 9), we conducted an extensive analysis of standard physiological plant growth parameters such as total chlorophylls, carotenoids, phenols, and flavonoids. Notably, the comprehensive profiles of these parameters revealed consistent increments in both wheat and tomato seedlings subjected to the respective treatments (Figure 10). The observed augmentation in photosynthetic pigments is of particular significance, as it directly contributes to the overall physiological robustness of the plants [60]. Enhanced levels of phenolic compounds signify a crucial defense mechanism against abiotic stress factors. These phenols effectively neutralize reactive oxygen species (ROS) and actively engage in antioxidative processes [61]. This defensive response was prominently evident in both wheat and tomato seedlings treated with either AS or BM, exhibiting a substantial increase in total phenolic content compared to the control group. Furthermore, the accumulation of total flavonoids emerged as an additional dimension of enhanced plant stress resilience. Flavonoids, as specialized secondary metabolites, play a pivotal role in countering a diverse array of plant stressors [62]. The substantial elevation of total flavonoid levels in our experimental wheat and tomato seedlings underlines the potential contribution of AS and BM treatments in fortifying these plants against various environmental challenges. These findings collectively underscore the positive impact of AS and BM treatments on the physiological growth profiles of both wheat and tomato seedlings. The observed increments in photosynthetic pigments, phenolic content, and flavonoids reflect a multi-faceted response, indicative of enhanced physiological vigor and stress resilience. These insights further elucidate the potential mechanisms through which AS and BM treatments contribute to the augmentation of plant growth and stress adaptation, contributing to their potential utility as effective bioinoculants or biofertilizers in sustainable agricultural practices.

These findings prompted field testing to further validate these notions with fruit harvest and other plant growth parameters. PGP effects resulted in overall enhanced wheat and tomato harvests (Figure 13 and Figure 15). The productivity enhancement traits were relatively more prominent with the BM isolate as seen under various treatments. At the outset, this study is the first to report on any PGP microbial endophyte from *E. prostrata*.

As previously emphasized, certain *L. amingena* strains have been associated with pathogenic attributes, albeit with minimal severity of infections that are unlikely to contribute to disease [59,65,72,73,74,75,76,77]. While debates persist regarding the hygienic relevance of *Lelliottia* strains, their genomes stand devoid of conventional virulence factors like the type III secretion system, cytotoxins, or hemolysins [59]. The study extensively delves into genomic analyses, with a specific emphasis on the microbial strains’ hygienic implications and adaptations to oligotrophic environments. Moreover, it delves into the intricacies of a model elucidating the proliferation mechanism [59].

It is not surprising that many of the isolated strains which show general features of a PGPB may confer pathogenicity in humans, other animals, or plants [90]. As we consider advancing toward the commercial application and translation of these PGPBs (AS/BM) in bioinoculant and biofertilizer formulations, it is imperative to conduct meticulous pathogenesis and toxicity profiling. In line with our own in vitro investigations involving wheat and tomato seeds, we have successfully validated the PGP potentials of our AS/BM isolates by utilizing their cell-free spent supernatants. This innovative approach circumvents direct bacteria–plant interactions. We aim to harness this method to develop and evaluate cell-free biofertilizer formulations, mitigating both practical and clinical concerns. Exploring these translational research opportunities with cell-free formulations of AS and/or BM, along with potential consortia, not only presents a promising avenue for sustainable agriculture and enhanced crop productivity but also underscores the importance of addressing safety and regulatory concerns before widespread application. This strategic approach aligns with the contemporary paradigms of agri-biotechnology, ensuring that innovation is accompanied by meticulous evaluation and consideration of potential implications.

Limited research has been conducted to ascertain the potential colonization, dissemination, and trafficking abilities of Lelliotia spp. (both plant-growth-promoting and Pathogenic strains) within plant hosts. Additionally, it remains unclear whether supplementing soil or seeds with these bacteria would yield consistent and reproducible delivery of plant-growth-promoting (PGP) traits to cultivated plants. Pertinent questions arise regarding the endophytic potential of these bacteria in cultivated plants—can they establish as endophytes or are they confined to the rhizosphere and soil? Furthermore, are endophytes from succulents and extreme habitats endowed with distinct advantages over other endophytic bacteria? We plan to address these queries with multi-faceted investigations on our *Lelliotia amnigena* isolates (AS/BM) in different ecological contexts. Such research is essential for advancing our understanding of these bacteria’s roles in plant–microbe interactions and their potential applications in sustainable agriculture. We further wish to explore the further avenues where PGP microbial isolates would allow bio-tapping biotechnological outputs from the otherwise ignored resilient spurge species.

## 4. Materials and Methods

### 4.1. Euphorbia Prostrata (EP) Sampling, Processing, and In Vitro Establishment

All local, national, and international guidelines and legislation were adhered to while collecting and culturing EP plants in this study. Healthy whole plants of *Euphorbia prostrata* Aiton (EP) were sampled in the hot summers (of June 2022) from various locations within the Chandigarh University campus, in the vicinity within and/or near new construction sites heavily concentrated with cement mixtures and sands on pedestrian pavements. Plants were identified as EP based on their floral characteristics well distinguished from *E. maculate*, *E. serpens*, and/or other similarly looking spurges [56,57,58]. Shoot and root segments were trimmed, and we proceeded with our previously standardized in vitro surface sterilization protocol for succulent plants [49,51] with slight modifications. In brief, plant material was washed under running tap water for 1 h followed by rinsing with Tween 20 froth for 5 min and 0.5% Dettol solution. Later, the plant material was surface-sterilized under a laminar hood by treatment with a 0.1% solution of mercuric chloride (45 s), followed by 70% ethanol (1 min) and three subsequent washes (3 min each) with sterile distilled water (SDW). Internodal segments (1–1.5 cm^2^) were trimmed and inoculated readily over semi-solid (with 0.8% agar) Murashige and Skoog media [91] supplemented with 3% sucrose and 0.8% agar (MSA medium). The final pH of MSA was set to 5.88 before autoclaving. Cultures were incubated in controlled PTC room conditioning (22  ±  2 °C temperature, 60 to 65% rel. humidity, and 16:8 hr light/dark photoperiods).

### 4.2. Isolation of Bacterial Endophytes

EP in vitro PTC trials followed after above-surface sterilization was routinely observed on a long term (3–4 weeks post in vitro establishment) for any microbial growth emanating from the EP explants and specifically not affecting in vitro response from EP. Any contamination from within 3–4 weeks of in vitro EP establishment or contamination deemed to occur from a source other than the explants was discarded and not considered in the study. When apparent, a loopful of culture or colonies with limited growth around the explant (few colonies) which could be picked using a sterile tooth-pick were streak-plated over a nutrient agar plate (incubated overnight at 37 °C) for colony phenotyping under a light microscope (Olympus CH20i, Haryana, India). Single colonies were then subcultured further onto fresh NA plates and were later used to inoculate 5 mL sterile nutrient broth (NB) for overnight growth (at 37 °C, 150 rpm, dark) for preparation as glycerol stock for culture storage backups and further analyses when needed.

### 4.3. Morphological Analyses

Bacterial isolates were morphologically characterized based on visual observations of colony growth and behavior over the NA plate, followed by direct close examination under a light microscope (Olympus CH20i) and also using SEM (detailed below). Similarly, plant (wheat and tomato) seeds/seedlings and/or full-grown plants resulting from the various in vitro/ex vitro/field treatments with/without bacterial isolates (discussed below) were also recorded for morphometrics around shoots, roots, their branching, and root hair density using visual observations and/or close examinations under a stereomicroscope (Nikon-745T fitted with a 5 Megapixel digital camera, Nikon, Mumbai, India).

### 4.4. Scanning Electron Microscopy

Cell surface morphology of microbial isolates was also studied using field-emission scanning electron microscopy (Fe-SEM) at Punjab University, Chandigarh. For this purpose, bacteria in their log phase were collected (by centrifuging at 7000 rpm, 4 °C, for 5 min). The resultant pellet was subsequently washed twice with phosphate-buffered saline (1X PBS, pH 7.2). This was followed by overnight fixation with 2.5% glutaraldehyde (at 4 °C) and three consecutive washes with 1XPBS. The samples were then dehydrated under stepwise treatment with an ethanol gradient (10 to 100% final concentration). The pellet was then dried, sputter-coated with gold, and observed on Fe-SEM equipment [92].

### 4.5. Biochemical Methods and Assays

Biochemical assays such as Gram’s staining and tests for catalase, methyl red, indole, citrate utilization, Voges–Proskauer, starch hydrolysis, urease, oxidase, nitrate reduction, motility, H_2_S production, tween 20/80 hydrolysis, α-ketolactose utilization, carbohydrate utilization, and NaCl tolerance were performed, most of which followed recommendations of standard protocols [93].

### 4.6. Enzyme Activity Assays

Cellulolytic activity in bacterial isolates was determined by detecting cellulose degradation. The freshly grown culture was spot-inoculated on carboxymethyl cellulose (CMC) agar plates and incubated at 37 °C for 24 h. Following this, the media were flushed with an iodine solution. Positive results were indicated by a clear halo zone [94]. Proteolytic activity was analyzed by spot inoculating bacteria on skim milk agar (Himedia, Mumbai, India) which would show a clear halo zone after overnight incubation in the dark at 37 °C [95]. Similarly, lipase activity was assessed by a clear halo zone after spot inoculation on a tributyrin agar base (supplemented with 1% Tributyrin) and incubation at 37 °C (for 24–48 h) [96]. Similarly, a halo zone after spot inoculation of the bacteria over Pectinase Screening Agar Medium (PSAM) (incubation at 37 °C for 72 h) indicated a positive pectinase activity [96]. Finally, amylase activity was also assessed with a yellow halo zone over starch agar spot-inoculated with bacteria incubated at 37 °C for 24–48 h, followed by a plate flooded with 1% iodine (for 20 min) [97].

### 4.7. Bacterial Motility Test

Two standard methods were employed to conduct the motility test [98]. The first method involved using semi-solid agar and the wet mount method. In the former, bacteria were stabbed vertically deep into an agar butt (SIM Medium Butt; Himedia, Mumbai, India) using a fine loop. The agar butt was then incubated overnight at 28 °C, following the manufacturer’s recommendations. The second method consisted of preparing a wet mount of bacteria on a glass slide, which was then observed for growth patterns under a light microscope (Metzer, Vision plus-5000 DPCT). For both methods, fresh inocula were obtained from a single colony on NB media.

### 4.8. Antibiotic Activity Assays

The bacterial isolate’s antibiotic sensitivity was assessed using a standard disc diffusion test following the CLSI guidelines [99,100]. To begin the assay, a bacterial starter culture was raised overnight from a single colony in NB at 28 °C and 200 rpm. The culture was then spread-plated on Muller Hinton agar (MHA; Himedia, Mumbai, India). After approximately 30 min of inoculum soaking, susceptibility discs containing various antibiotics (Himedia) were placed on the plates. These plates were subsequently incubated overnight in the dark at 28 °C. The extent of bacterial susceptibility to the antibiotics was determined by measuring the observed zone of inhibition, which represented the diameter of the region where bacterial growth was inhibited. A larger zone indicated greater susceptibility, while a smaller or absent zone indicated antibiotic resistance. The results were expressed as the means of the means obtained from three replicates, with each replicate stemming from three trials per antibiotic tested.

### 4.9. Molecular Characterization of Bacterial Isolates

The bacterial isolates were subjected to genomic DNA isolation using a kit protocol from Himedia, India, to identify their respective 16S rRNA sequences. This identification was carried out through PCR amplifications of the genomic DNA using universal primers 27F: AGAGTTTGATCMTGGCTCAG and 1492R: TACGGYTACCTTGTTACGACTT. The PCR reactions were composed of 200 mM PCR buffer, 1.5 mM MgCl_2_, 10 pmol µL^−1^ of each primer (Bioserve Biotechnologies India Pvt. Ltd., Hyderabad, India), 10 mM dNTPs, 5 units of Taq-DNA polymerase (Himedia, Mumbai, India), and molecular-biology-grade water (Himedia, Mumbai, India) to a total volume of 50 µL per reaction. The PCR program, run on a BIORAD S1000™ thermal cycler, included the following steps: an initial denaturation at 95 °C for 5 min, followed by 32 cycles, each comprising intermittent denaturation at 95 °C for 1 min, annealing at 54 °C for 1.5 min, and extension at 72 °C for 1 min. The program concluded with a final extension step at 72 °C for 10 min. The resulting amplicons were then extracted from the gel using the Himedia gel extraction kit and ligated to the pGEM-T easy vector from Promega, New Delhi, India. The ligated vectors were transformed into DH5-α-competent bacteria using the heat-shock protocol. To screen for recombinant clones, a blue–white screening method was employed, and the presence of inserts in the plasmids was verified using colony PCR and/or restriction enzyme digestions. Plasmid isolates with the desired gene inserts were sequenced using universal M13 primers from Eurofins Genomics India Pvt., Bangalore. The obtained sequences were screened and trimmed to remove vector backbone sequences using the VecScreen tool (https://www.ncbi.nlm.nih.gov/tools/vecscreen/; accessed on 4 January 2023) and then analyzed for chromatograms using CHROMAS software (version 2.6.6, Technelysium Pty Ltd, South Brisbane QLD, Australia; https://technelysium.com.au/wp/chromas/; accessed on 4 January 2023). For sequence similarity searching, BLAST was performed using GenBank (https://www.ncbi.nlm.nih.gov/genbank/; accessed on 4 January 2023) and EzBioCloud databases (https://www.ezbiocloud.net/; accessed on 4 January 2023). The Clustal W program was utilized to align highly similar sequences, and phylogenetic trees based on various algorithms were constructed using MEGA software (version 11.0.11, https://www.megasoftware.net/downloads/dload_win_gui; accessed on 9 January 2023) [101]. All procedures involving the kit followed the manufacturer’s recommendations.

### 4.10. PGP Activity Assays over Bacterial Isolates

Many standard assays that indicate plant-growth-promoting (PGP) attributes in bacterial isolates were carried out as detailed below.

#### 4.10.1. Potassium Solubilization Activity

The potassium solubilization ability of the bacterial isolate was assessed using a standard spot plate assay on Aleksandrow agar media (Himedia, Mumbai, India). The assay involved inoculating the agar with a loopful of an overnight-grown bacterial culture and then incubating it at 28 °C. Positive potassium solubilization was confirmed by the presence of clear zones around the bacterial colonies.

#### 4.10.2. Phosphate Solubilization Activity

The bacterial isolates were evaluated for phosphate solubilization using a standard Pikovskaya’s agar plate protocol with minor adjustments [102]. Specifically, an isolate was spot inoculated on Pikovskaya’s agar (Himedia, Mumbai, India) containing tricalcium phosphate as an insoluble source of phosphorus. The culture was then incubated at 28 °C for a maximum of 10 days, allowing time for the development of a clear halo around the bacterial colonies. The presence of this halo indicated positive phosphate solubilization, which can be quantified using the following formula:

$$\begin{array}{c} Phosphate\;solubilization\;index\;(cm)\\ \;\;\;\;\;\;\;=\frac{Colony\;diameter\;\left(cm\right)+Halozone\;diameter(cm)}{Colony\;diameter\;(cm)} \end{array}$$

#### 4.10.3. ACC Deaminase Activity

The presence of ACC (1-aminocyclopropane-1-carboxylic acid) deaminase activity in a bacterial isolate was assessed by cultivating it on DF minimal salt medium supplemented with 2 g/L (NH_4_)_2_SO_4_ and incubating it for 72 h at 28 °C [103]. ACC deaminase is an enzyme that plays a crucial role in reducing ethylene levels in plants when they are under stress. It achieves this by breaking down ACC, which is a precursor of ethylene synthesis, into ammonia and α-ketobutyrate.

#### 4.10.4. Siderophore Production

For screening and assessing siderophore production, a freshly prepared Chrome Azurol S (CAS) reagent was utilized. Before the test, all cultures and test vessels were treated to remove any trace amounts of iron. This was achieved by rinsing them overnight in 3 mol L^−1^ HCl and then washing them with sterile distilled water (SDW) [104]. The qualitative test for siderophore production followed the standard CAS agar plate method [105]. In this procedure, nutrient agar (NA) plates were supplemented with 10% CAS reagent. The bacterial isolate was streaked onto these plates, and then the plates were incubated at 28 °C for a week. A positive result for siderophore production is indicated by the formation of a yellow-to-orange halo around the streaked bacterial colonies on the CAS agar plates. This halo indicates the presence of siderophores, which are iron-chelating compounds secreted by bacteria to scavenge and solubilize iron for their growth and survival.

#### 4.10.5. IAA Production

To determine indole-3-acetic acid (IAA) production, a standard method [106] was employed with slight modifications. The bacterial isolates were cultured in nutrient broth (NB) supplemented with 0.1% tryptophan and then incubated for 10 days at 28 °C and 120 rpm. Every two days, culture aliquots were withdrawn and centrifuged to remove the cell pellet (centrifugation was performed at 10,000 rpm for 15 min). The resulting supernatant (1 mL) was mixed thoroughly with 2 mL of freshly prepared Salkowski reagent (consisting of 1 mL FeCl_3_ and 50 mL 35% HClO_4_) [107]. The mixture was allowed to stand in the dark for 30 min. The presence of pink coloration in the test samples indicated IAA production. To quantify the amount of IAA, spectrophotometric measurements were taken at a wavelength of 530 nm (OD_530_). A standard curve was constructed using commercially purchased IAA (Sigma-Aldrich, USA), which served as a reference for quantification.

#### 4.10.6. Ammonia Production

Ammonia production was investigated following a standard protocol [93]. The bacterial isolates were inoculated in peptone water from Himedia, Mumbai, India, and then incubated for 10 days at 28 °C and 120 rpm. At different time intervals, test aliquots were withdrawn and subjected to centrifugation to remove the cell pellet. Centrifugation was performed at 10,000 rpm for 15 min. Then, 1 mL of the supernatant was mixed with 50 µL of Nessler’s reagent from Thermofisher Scientific, New Delhi, India. The appearance of a yellow-to-brown coloration in the test aliquots indicated positive ammonium production. To quantify the amount of ammonium, spectrophotometric measurements were taken at a wavelength of 450 nm (OD_450_). A standard curve was constructed using commercially purchased ammonium sulfate from Himedia, Mumbai, India, which served as a reference for quantification.

#### 4.10.7. Gibberellic Acid (GA) Production

The production of gibberellic acid (GA) by bacterial isolates was determined using a standard spectrophotometric assay [108]. Briefly, culture supernatants were collected at various time intervals after centrifugation to remove cell debris. The supernatants were mixed with zinc acetate and potassium ferrocyanide solutions, followed by a second centrifugation step. The resulting supernatant was then mixed with HCl and incubated. Absorbance was measured at 254 nm (OD_254_) using a spectrophotometer against a blank containing 5% HCl. The absorbance values were then compared to a standard curve constructed with commercially purchased GA (ranging from 20 to 200 μg mL^−1^, Himedia, Mumbai, India) to quantify the amount of GA produced by the bacterial isolate.

#### 4.10.8. Zinc Solubilization Assay

The preliminary test used a standard plate assay [109] with the bacterial isolates spot-inoculated on a semi-solid basal media mixed with 0.1% zinc oxide (insoluble, Himedia, Mumbai, India). The plates were then incubated at 28 °C for a week. After the incubation period, any clear zones around the spot colony indicated zinc solubilization activity. An atomic absorption spectrometry (AAS) method [109] was employed for the quantification of zinc solubilization. The bacterial isolates were inoculated into the broth (media as described above), from which samples were withdrawn at various time intervals and then centrifuged at 10,000 rpm for 15 min to obtain the supernatants. The supernatants were then fed to a Perkin Elmer AAS instrument at Oxygen Labs Pvt. Ltd. (Baddi, Himachal Pradesh, India) for analysis.

#### 4.10.9. Nitrogen Fixation

The bacterial isolates’ nitrogen-fixing ability was assessed using a standard method based on their growth on nitrogen-free media [110]. In this method, the bacterial isolate was streaked on Jensen media and incubated at 28 °C for a week. Positive nitrogen fixation was indicated by the growth of bacteria with glistening colonies and/or streaks on the nitrogen-free media.

#### 4.10.10. Hydrogen Cyanide Production

The production of hydrogen cyanide by a bacterial isolate was determined using a standard method [111]. For this test, the bacterial isolate was streaked over nutrient agar (NA) plates supplemented with glycine (4.4 g/L). An autoclaved Whatman filter paper disc (No. 1) soaked in 2% sodium carbonate solution (prepared in 0.5% picric acid) was placed on the plate near the streaked bacteria. The plates were then incubated in the dark at 28 °C for 4–5 days. The development of a dark orange-to-red coloration on the filter paper indicated positive hydrogen cyanide production by the bacterial isolate.

#### 4.10.11. Biofilm Formation

The biofilm formation ability of the bacterial isolates was evaluated using the Congo red agar (CRA) plate method [112], which is a qualitative screening method for biofilm formation by microorganisms. After streaking the bacterial isolates on CRA (0.8 g Congo red, 50 g sucrose with 37 g brain heart infusion media mixed for a liter preparation and gelled with 15 g agar; pH 7.2), the plates were incubated at 28 °C for 24 h. Positive biofilm formation was indicated by crystalline blackening of colonies with a dry consistency.

### 4.11. Seed-Priming Treatments with Bacterial Isolates

As per the need of various trials, known quantities of seeds of the commercially available varieties of wheat (UNNAT PBW 343) and tomato (Sakura F1 super Arjun) were purchased from a local seed vendor (Kharar, Mohali, Punjab, India) and sorted for picking healthy seeds lots using a commonly manifested approach. This involved swirling them under tap water and rejecting any floating seeds from the lot(s). Seeds were washed thoroughly thrice (2 min each) with ample froth of a mild detergent solution (froth of two drops of Tween-20 in 50 mL SDW) to clear dirt and dust. Further, seeds were rinsed thrice with ample SDW for about half an hour and then soaked overnight in ultra-filtered tap water (TW) for imbibition. The next day, a set of seeds were either primed with clarified spent culture supernatants of overnight grown bacteria (exclusively for in vitro trials with seeds) and/or primed (in case of ex vitro/field trials, see below) by co-cultivation with an appropriate volume of overnight grown culture (1 × 10^8^ CFU mL^−1^) of the bacterial isolate(s) (raised from a single colony on NA, later subcultured overnight over NB; incubated at 37 °C, 150 rpm, dark). Co-cultivation conditions were kept almost the same as those for bacterial incubations (37 °C, 120 rpm, dark). The control set saw treatment with only NB devoid of any bacterial isolates or their spent culture supernatants. Both bacterial culture and priming media contained ampicillin (34 mg/L) as a selection agent.

### 4.12. In Vitro and Ex Vitro Validation of PGP Effects on Commercial Crops

Wheat and tomato seeds were established in vitro over MSA in sealed jam jars (3 seeds per jar) and incubated under control PTC room conditioning detailed before. Observations for seed germination response and seedling growth parameters were recorded every day for both primed and control treatments. The experiment was repeated thrice with five replicates each time. Similarly, ex vitro validation of PGP effects from the bacterial isolates on wheat and tomato was carried out following a completely randomized design (CRD) in controlled glasshouse conditions (25–27 °C, 70–90% relative humidity, and natural photoperiods). In brief, primed and/or control seeds were sown over soil beds in plastic trays (flat trays with dimensions of 43 × 34 × 7 cm for wheat trials and tomato trials, nursery potting trays containing 50 mini pots (each with dimensions of 4 *×* 4 cm) were employed). Soil beds in respective trays (both wheat and tomato) covered 4 cm in height and were prepared with mesh-sorted and autoclaved garden soil without any pretreatment with any fertilizer or biocidal agent. Seeds were sown at a depth of 2 cm in these soil beds and were spray-watered on the same day post-sowing (with ca. 500 mL TW per tray for wheat trials and 200 mL per tray in case of tomato trials). Thereafter every day followed a consistent spray-watering regime of 150 mL for the wheat and tomato trials. This included once-per-week booster dosing of the soil beds in seed-priming-treated crop tray sets with fresh overnight-NB-raised bacteria (1 × 10^8^ CFU ml^−1^) once per week, while control beds were treated similarly with NB. Seed germination and seedling growth profile were monitored and recorded periodically. After a fixed trial length, in each of the tomato and wheat trials, followed the drain-out assessments of various growth parameters such as the morphometrics of shoots, roots, their branches, root hair density, and overall fresh and dry weight (FW and DW, respectively). In a separate extended trial with tomato seed treatments, when the average height in control treatments was thrice (≥12 cm) the size of tray pots, each seedling was transferred individually to big (of 1.5 L volumetric capacity) plastic pots. This allowed consistent recording of shoot morphometrics beyond the drain-outs.

### 4.13. Field Trials and Productivity Profiling

To further validate the ability of seed-priming-assisted plant growth promotion and more importantly to test its translational outcome in the productivity enhancement of wheat and tomato crops in near-real field settings, small controlled field trials were conducted following a CRD in a well-equipped polyhouse facility at university farms in liaison with the University Institute of Agricultural Sciences. In brief, and as mentioned before, primed and control-treated seed lots were sown at a depth of 2 cm over ca. 9-inches-raised soil beds (without any pretreatments with fertilizers or biocides). Watering and/or booster-dosing volumes were proportionate to those carried out under ex vitro trials but used the soil drenching method instead of spray maneuvers. Observations for seed germination percentage were carried out at a two-day interval. Morphometric recordings were conducted a week after seed sowing (later on every weekly basis) with seedling maturation. Harvest data were accordingly recorded in the laboratory following the pulling out of wheat crops in one instance after complete inherent drying of shoots occurred for all replicates in the control treatment sets. Similarly for tomato harvest, pruning of all tomato fruit bunches was carried out readily when almost all fruits in either seed-priming or control sets turned red. Based on the area of plots used to carry out field trials for each experimental set with wheat and tomato crops under these small-scale field settings, estimates were hypothesized for yield from an acre plantation. Weedy outgrowths in all experimental sets when observed were carefully removed by hand pruning at weekly intervals before any irrigation and/or booster dosing was conducted.

### 4.14. Plant Physiological Growth Profiling

Physiological growth profiling was carried out on wheat and tomato seedlings under ex vitro trials to ascertain the influence of seed priming intervention with the bacterial isolates. These involved estimating phenolic compounds, flavonoids, and photosynthetic pigments in the studied plant leaf samples following standard methods [113,114,115]. In brief, the total phenolic content (TPC) of the samples was determined using spectrophotometry with Folin Cicocalteu reagent. A 500 µL aliquot of the extract solution was mixed with 2.5 mL of Folin Cicocalteu reagent (10% *v*/*v*). Then, 2 mL of 1M Na_2_CO_3_ was added, and the mixture was incubated in the dark for 2 h. After incubation, the absorbance of the resulting dark blue solution was measured at 760 nm. Gallic acid was used as a standard for calibration, and the TPC results were expressed in µg gallic acid equivalents per gram (µg GAE/gm) of the sample. The total flavonoid content (TFC) was estimated based on the reaction between flavonoids and aluminum chloride (AlCl_3_), which forms a colored complex. For the estimation, a 500 µL extract of the sample was mixed with 1.5 mL of methanol. Then, 0.1 mL of AlCl3 (10%) and 0.1 mL of sodium acetate solution (1M) were added to the mixture, followed by the addition of 2.8 mL of water. The mixture was incubated in the dark for 30 min, and the absorbance was measured at 430 nm. Quercetin was used as a reference for calibration, and the TFC results were expressed in µg quercetin equivalents per gram (µg QE/gm) of the sample. For the estimation of chlorophyll a and b and carotenoid content, 1 gm of leaf sample was crushed in a mortar and pestle with 20 mL of 80% acetone and 0.5 mg of MgCO_3_ powder and then stored at 4 °C for 2 h. After centrifugation at 500 rpm for 5 min, a 0.5 mL aliquot of the supernatant was mixed with 4.5 mL of acetone, and the absorbance was measured at 663 nm, 645 nm, and 450 nm to calculate the concentrations of chlorophyll a, chlorophyll b, and carotenoids, respectively.

### 4.15. Statistical and Computational Approaches

All tests were performed at least thrice with three or more replicates per trial. Data values and standard error in graphs, depict the mean of the values and standard deviations, respectively from replicates of three or more trials. In bar charts, for all variables with the same letter over the bars, the difference between the means is not statistically significant; otherwise, if two variables with bars have different letters, they are significantly different (P ≤ 0.05) as derived using the Tukey’s test. For DNA barcoding analyses, widely used software suites and/or programs and online applications, namely CLC workbench (version 6.5.1), DECIPHER (version 2.19.2) [116], and VECSCREEN (https://www.ncbi.nlm.nih.gov/tools/vecscreen; accessed on 4 January 2023), were employed. Molecular-level identification of bacterial isolates used 16S rRNA sequencing results which were processed for similarity search using the GenBank (NCBI) (https://www.ncbi.nlm.nih.gov/genbank; accessed on 4 January 2023) and EzBioCloud (https://www.ezbiocloud.net; accessed on 4 January 2023) databases and were performed using BLAST and EzBioCloud tools, respectively. To align highly similar sequences and construct phylogenetic trees, the Clustal W program and MEGA (version 11.0.11, https://www.megasoftware.net/downloads/dload_win_gui; accessed on 9 January 2023) [101] software were used. The phylogenetic tree was built using the neighbour-joining (NJ) method, with 1000 bootstrap replications. The evolutionary distances between the closely related bacterial strains were computed using the maximum composite likelihood (MCL) method. Moreover, to assess the phylogenetic relationship between the bacterial isolates projected in this study and other closely related species, several algorithms including maximum parsimony, maximum likelihood, minimum evolution, and UPGM were also employed. The 16S rRNA nucleotide sequences of the bacterial isolates (EPAS and EPBM) documented in this study were deposited in the GenBank database under the accession numbers OR342320 and OR342321, respectively. Figures were prepared in MS Excel and MS PowerPoint (Microsoft Office Home and Student 2019; Version 2307).

## 5. Conclusions

In the context of stress biology, ground-dwelling spurges have gained attention due to their unique abilities to tolerate and adapt to stressful environmental conditions, including arid and semi-arid regions, saline soils, and polluted sites. And from the viewpoint of biotechnology, they are emerging as focal points for translational research into phytoremediation, bioenergy production, and phytomedicines. Our research endeavors focused on exploring the potential of endophytic microbial communities associated with resilient succulent plants, particularly ground-dwelling spurges of the Euphorbiaceae family. Our investigation led to the isolation of two distinct *Lelliotia amnigena* isolates, AS and BM, from the internodal explants of the sandmat spurge, *Euphorbia prostrata*. Despite their initial categorization as potential contaminants, these isolates exhibited remarkable plant-growth-promoting (PGP) properties, resulting in enhanced growth and productivity of wheat and tomato crops under ex vitro conditions. Notably, the PGP effects were more pronounced with the BM isolate and were further accentuated through seed-priming treatments. Our findings provide evidence of the potential for AS and BM isolates to serve as plant endophytes, as they were shown to enhance in vitro plant regeneration efficiency in their latex-bearing spurge host. Notably, the observed growth enhancements extended to physiological growth parameters, such as chlorophylls, carotenoids, phenols, and flavonoids, suggesting a multi-dimensional improvement in plant stress resilience and physiological vigor.

The productive outcomes observed in ex vitro trials prompted further evaluation through field testing, which substantiated the potential of AS and BM isolates to enhance crop productivity in wheat and tomato. While *L. amnigena* strains have been associated with pathogenesis in certain contexts [59,65,67,72,73,74,75,76,77,78,79,80], they generally exhibit limited virulence factors [59]. As we contemplate the translation of our PGPB findings into practical applications, careful pathogenesis and toxicity profiling remain essential steps. Our approach of utilizing cell-free spent supernatants of AS and BM isolates presents an innovative strategy for developing biofertilizer formulations while addressing safety concerns.

Crucially, our investigation has unveiled intriguing questions regarding the colonization, dissemination, and endophytic potential of *Lelliotia* spp. in cultivated plants. The complex interplay between these bacteria and their hosts, along with the exploration of translational opportunities, underscores the need for rigorous and comprehensive research. By harnessing the bioprospects from resilient plants and their associated microbial communities, we strive to pave the way for sustainable agriculture while concurrently tapping into the unexplored potential of these often overlooked and robust plant species. Such translational research movements are highly demanded considering the global zero-hunger target to be achieved by 2030 with impeding scenarios of climate change and the other Sustainable Development Goals (SDGs) of the United Nations Organization (UNO) [117].

## Figures and Tables

**Table 1:** Biochemical, morphological characterizations, and enzyme activity assay with the EP isolates.

*Assay Classes*	*Activity/Assays*	*Characteristics*	
***Morphology and Growth Responses***		AS	BM
	Gram reaction	-ve	-ve
	Shape in LM and SEM	Rod-shaped	Rod-shaped
	Colony on NA	Smooth, irregularly shaped, pale white, and motile	Clustered motile, irregularly shaped, pale white
	Colony on MSA	Smooth and white	Smooth and white
	Colony on LBA	Smooth, irregularly shaped, off-white, and motile	Clustered motile, irregularly shaped, yellowish-brown
***Standard biochemical responses***			
	Catalase test	+	+
	Methyl red	+	+
	Indole test	+	+
	Citrate utilization	+	+
	Voges Proskauer	-	-
	Starch hydrolysis	-	-
	Urease test	-	-
	Oxidase test	-	-
	Nitrate reduction	-	-
	Motility test	+	+
	Hydrogen sulfide test	-	-
	Tween-20 hydrolysis	-	-
	Tween-80 hydrolysis	-	-
	α-ketolactose utilization	-	-
***Carbohydrate utilization***			
	Glucose	-	**+**
	Sucrose	+	+
	Starch	-	-
	Mannitol	-	-
	Lactose	+	+
	Dextrose	+	+
	Fructose	-	-
	Gelatin	-	-
	Arabinose	-	-
	Adonitol	-	-
	Sorbitol	-	-
	Rhamnose	-	-
***Growth in NaCl***			
	0%	+	+
	1%	+	+
	2%	+	+
	3%	+	+
	4%	+	+
	5%	+	+
***Enzyme activities***			
	Cellulase	-	-
	Protease	-	-
	Lipase	-	-
	Pectinase	-	-
	Amylase	-	-

**Table 2:** **Antibiotic sensitivity of AS and BM isolates.** Disc size = 6 mm; S = less susceptibility (7–10 mm); S^+^ = susceptibility (11–20 mm); S^++^ = high susceptibility (21–30 mm); S^+++^ = extreme susceptibility (31–40 mm); R = resistant (0 mm). All antibiotics were purchased from Himedia (Mumbai, India) with indicated catalog numbers (*Cat#*) in the first column.

*Cat#*	*Antibiotic (Concentration)*	AS		BM	
		*Inhibition Zone (mm)*	*Response*	*Inhibition Zone (mm)*	*Response*
SD039	Trimethoprim (5 μg)	30 ± 0.32	S^++^	25 ± 0.15	S^++^
SD031	Streptomycin (10 μg)	0	R	0	R
SD181	Spectinomycin (10 μg)	27 ± 1.23	S^++^	35 ± 0.8	S^+++^
SD028	Penicillin G (10 units)	0	R	0	R
SD133	Tetracycline (10 μg)	12.5 ± 0.45	S^+^	16 ± 0.16	S^+^
SD006	Chloramphenicol (30 μg)	0	R	0	R
SD016	Gentamicin (10 μg)	17.5 ± 0.15	S^+^	27.5 ± 0.25	S^++^
SD002	Ampicillin (10 μg)	0	R	0	R

**Table 3:** **Plant growth promotion assays with AS and BM isolates.** Various standard assays were carried out to infer plant growth promotion properties in both isolates. Periodic observations were recorded following standard assays, and the most significant effects are emphasized in bold. The values presented are the means of three replicates conducted in each of the three separate trials.

*PGP Traits*	48 h		96 h		144 h		192 h		240 h	
	AS	BM	AS	BM	AS	BM	AS	BM	AS	BM
**Potassium solubilization**	-	-	-	-	-	-	-	-	-	-
**Phosphate solubilization index (cm)**	-	-	-	-	-	-	**1.25 ± 0.25**	-	-	-
**ACC deaminase**	-	-	-	-	-	-	-	-	-	-
**Siderophore %**	-	-	-	-	-	-	-	-	-	-
**IAA (μg/mL)**	18.06 ± 0.36	20.35 ± 0.11	15.29 ± 0.49	38.16 ± 0.20	28.02 ± 1.66	**79.49 ± 0.33**	37.70 ± 0.25	**88.50 ± 0.33**	33.29 ± 0.45	74.42 ± 1.69
**Ammonia production (µmol/mL)**	18.78 ± 1.02	24.16 ± 1.45	24.12 ± 0.29	26.18 ± 0.55	**41.52 ± 0.83**	**42.67**	29.08 ± 2.21	33.19 ± 1.05	28.92 ± 0.45	36.54 ± 0.19
**Giberellic acid (μg/mL)**	35.23 ± 0.35	40.09 ± 0.36	48.59 ± 0.79	53.37 ± 0.85	**379.5 ± 0.1**	**452.34 ± 0.67**	288 ± 1.8	243.47 ± 0.19	69.86 ± 0.45	69.92 ± 1.6
**Zinc solubilization**	-	+	-	+	-	+	-	+	-	+
**Zinc solubilization (μg/mL)**	-	204.3	-	**274.4**	-	192.8	-	228.5	-	182.9
**N_2_-Fixation**	+	+	+	+	+	+	+	+	+	+
**HCN production**	-	-	-	-	-	-	-	-	-	-
**Biofilm production**	-	-	-	-	-	-	-	-	-	-

## Data Availability

The 16S rRNA gene sequence homologs for AS and BM isolates have been deposited in NCBI’s GenBank (accession numbers OR342320 and OR342321, respectively). Any raw data supporting the conclusion of this article will be made available by the corresponding authors, without undue reservation, upon formal request.
